# Selectively targeting BCL6 using a small molecule inhibitor is a potential therapeutic strategy for ovarian cancer

**DOI:** 10.7150/ijbs.86303

**Published:** 2024-01-01

**Authors:** Min Wu, Jiuqing Xie, Yajing Xing, Lin Zhang, Huang Chen, Bin Tang, Miaoran Zhou, Shiyi Lv, Dongxia Huang, Shuyi Jian, Cili Zhou, Mingyao Liu, Weikai Guo, Yihua Chen, Zhengfang Yi

**Affiliations:** 1 East China Normal University, Shanghai Key Laboratory of Regulatory Biology, Institute of Biomedical Sciences and School of Life Sciences, 500 Dong Chuan Rd, Shanghai 200241, China.; 2 The Jointed National Laboratory of Antibody Drug Engineering, Henan University, Kaifeng, 475004, China.; 3 Department of Gynecology, The Second People's Hospital of Wuhu, Wuhu, Anhui 241000, China.; 4 Shanghai University of Traditional Chinese Medicine, Shanghai, 201203, China.

**Keywords:** BCL6, Ovarian cancer, BTB domain, Metastasis

## Abstract

Ovarian cancer is one of the tumors with the highest fatality rate among gynecological tumors. The current 5-year survival rate of ovarian cancer is <35%. Therefore, more novel alternative strategies and drugs are needed to treat ovarian cancer. The transcription factor B-cell lymphoma 6 (BCL6) is critically associated with poor prognosis and cisplatin resistance in ovarian cancer treatment. Therefore, BCL6 may be an attractive therapeutic target for ovarian cancer. However, the role of targeting BCL6 in ovarian cancer remains elusive. Here, we developed a novel BCL6 small molecule inhibitor, WK369, which exhibits excellent anti-ovarian cancer bioactivity, induces cell cycle arrest and causes apoptosis. WK369 effectively inhibits the growth and metastasis of ovarian cancer without obvious toxicity *in vitro* and *in vivo*. meanwhile, WK369 can prolong the survival of ovarian cancer-bearing mice. It is worth noting that WK369 also has significant anti-tumor effects on cisplatin-resistant ovarian cancer cell lines. Mechanistic studies have shown that WK369 can directly bind to the BCL6-BTB domain and block the interaction between BCL6 and SMRT, leading to the reactivation of p53, ATR and CDKN1A. BCL6-AKT, BCL6-MEK/ERK crosstalk is suppressed. As a first attempt, our study demonstrates that targeting BCL6 may be an effective approach to treat ovarian cancer and that WK369 has the potential to be used as a candidate therapeutic agent for ovarian cancer.

## 1. Introduction

Ovarian cancer (OV) is considered one of the most lethal of female genital malignancy, with the highest morbidity and mortality among gynecological cancer. In 2022, there were 19880 new diagnosed cases of ovarian cancer and 12810 deaths worldwide^1^. When ovarian cancer is diagnosed with stage Ⅰ or Ⅱ, the 5‑year survival rate can reach 89% and 71%^2^. However, ∼70% of ovarian cancer patients have distant metastatic disease (stage III or IV) when diagnosed due to its asymptomatic at early stages^3^,^4^. The 5-year survival rate for these patients is less than 25%^5^. The main issues leading to poor survival in OV patients are metastasis and chemotherapy resistance^6^. Therefore, the development of innovative treatments for preventing acquired chemo-resistance or ovarian cancer metastatic is critically required.

With the improvement on understanding regarding the pathogenesis underlying ovarian carcinogenesis, molecular targeted therapy has made significant progress in the current treatment of OV. PARP is a ribose polymerase found in the nucleus of eukaryotic cells and play an important role in DNA break repair^7^. Inhibition of PARP blocks DNA repair in BRCA-mutated ovarian cancer cells, leading to apoptosis^8^. Olaparib and niraparib are FDA-approved PARP inhibitors mainly used to treat advanced ovarian cancer harboring BRCA mutations^9^,^10^,^11^. Although PARP inhibitors can partially extend PFS in patients with ovarian cancer, unfortunately, they do not extend OS^12^. Inhibiting angiogenesis is one of the options for treating ovarian cancer. Bevacizumab is an anti-VEGF antibody that was approved by the FDA in 2014 for the treatment of cisplatin-resistant recurrent epithelial OV in combination with cisplatin and paclitaxel^13^. The above therapies helped improve ORR and PFS, however, the OS is not significantly improvement^13^-^16^. In addition, patients with severe toxicities, when compared between Bevacizumab therapy and placebo groups. In recent years, immune checkpoint inhibitors have made great breakthrough in various cancers, and anti-CTLA4 antibody was approved for melanoma^17^, and anti-PD-(L)1 antibody were approved to treat melanoma, lung cancer, and renal carcinoma^18^. However, these drugs have a limited effect on ovarian cancer, with ORR of only 10-15% and the median duration of PFS is only six months^4^,^19^,^20^. To sum up, despite the continuous development of molecular targeted therapies in recent years, the OS of patients with OV has not been significantly improved. New alternative strategies and drugs with more efficacy and less toxic side effects are urgently needed to improve the OS of OV patients.

The transcriptional factor BCL6 is a member of the ZBTB family identified from lymphoma that plays a crucial role in regulating germinal center response and has gained prestige as an oncogene in human cancers via negatively regulating many genes, such as tumor suppressor p53^21^, DNA damage sensing and checkpoint genes ATR^21^, proliferation checkpoints genes CDKN1A and CDKN2K^22^-^24^. Thus, BCL6 is considered as a promising therapeutic target for the treatment of some malignancies, for instance, lymphomas^25^, glioblastoma^26^, breast cancer^27^. In particular, previous study revealed that high expression of BCL6 is positively correlated with the poor prognosis of patients with ovarian cancer^28^. BCL6 is associated with drug resistance, stress response and tolerance of tumor cells^29^. Cisplatin is currently an effective chemotherapy drug for the treatment of OV. In this article, we confirmed that cisplatin treatment can induce the expression of BCL6 in OV cells. Therefore, it is speculated that tumors can be synergistically inhibited by combining BCL6 inhibitors with chemotherapy drugs, which may provide some treatment options for patients with drug-resistant ovarian cancer or patients who are insensitive to chemotherapy drugs.

Now, a number of BCL6 small molecule inhibitors (including reversible and irreversible) and BCL6 degraders have been reported. However, most of them have not been evaluated through target engagement experiments and no evidence suggested that these inhibitors can repress normal mouse antibody responses *in vivo^30^-^35^*, only a few of them show some* in vivo* efficacy in repressing the BCL6 biological function such as 79-6^36^ and FX1^37^. Among, FX1 can inhibit the germinal center formation. However, even for FX1, its activity is still poor. In our previous work, we described a BCL6 inhibitor, which can suppress diffuse large B-cell lymphoma (DLBCL) growth *in vitro* and *in vivo^38^*. Nevertheless, all of these inhibitors are against DLBCL, BCL6 targeted therapy in ovarian cancer has not been explored. Here, we describe a small-molecule referred to as WK369, which acts through disturbed the interaction between BCL6 and SMRT in vitro and inhibited the BCL6 biological function in vivo. Meanwhile, WK369 suppressed the growth and migration of OV *in vitro*, and more importantly, it also showed good antitumor effects on cisplatin-resistant ovarian cancer cells. WK369 suppressed tumor growth, metastasis, and prolonged survival in mouse models of ovarian cancer. The mechanism research showed that it exhibits anti-tumor activities may be attributed to reactivate p53, ATR and CDKN1A, inhibit BCL6-AKT and BCL6-MEK/ERK cross-talk signaling pathway. Thus, as a BCL6 inhibitor, WK369 provides a potential approach for the treatment of ovarian cancer.

## 2. Material and Methods

### 2.1. Cell Culture

293T, Farage, ES-2, SKOV3, MCAS, CAOV3, OVCAR8, IGROV-1, and HaCaT cell lines were provided by ATCC. Cell culture conditions were: DMEM medium (Gibco) with 10% FBS (Invitrogen) and 1% penicillin-streptomycin (Invitrogen), and the cell culture incubator conditions were set to 5% CO2, 37°C.

### 2.2. Cell Viability Assay

MTS assay (Promega, USA). First, spread 8 x 10^3^ cells into 96-well plates, and different concentrations of compoundwere added 24 hours later and treated for 72 hours. Before testing, add MTS and incubate in a 37°C incubator with 1-2 h. At 490 nm the absorbance was measuredand. The drug combination determination method was the same as above, and CalcuSyn software was used to calculate CI values in data analysis. CI value > 1 indicates that the compound combination has an antagonistic effect, but CI value < 1 indicates that the compound combination has a synergistic effect.

### 2 3. Cell Apoptosis Assay

Add different concentrations of WK369 to ovarian cancer cells. After treating the cells for 24 hours, use trypsin digestion and centrifugation to harvest the cells. Use pre-chilled PBS to remove excess culture medium from the cells, and then resuspend them in 1×binding buffer. Add 50 μg/mL AV and 50 μg/mL PI to cell suspension, and stain for 15 minutes at 4°C. Flow cytometry (FACS Calibur, BD Biosciences) was used for analysis.

### 2.4. Cell Cycle Analysis

Add different concentrations of WK369 to ovarian cancer cells. After treating the cells for 24 hours, use trypsin digestion and centrifugation to harvest the cells. Use pre-cooled PBS to remove excess culture medium from the cells. Add pre-cooled 70% ethanol for the last time and incubate at 4°C overnight. The next day, use PBS to wash excess 70% ethanol in the cells, then add 20 μg/mL RNase next60 μg/mL PI to the cell suspension, and stain for 30 minutes at 4°C in the dark. Finally, flow cytometry was used for analysis.

### 2.5. Quantitative Real-Time PCR

Different concentrations of compounds were added to Farage, SKOV3 and ES-2 cells for 24 hours. After the treatment, excess culture medium was washed away with PBS, and TRIzol reagent (Invitrogen) was added to collect the cells and extract RNA. After detecting the concentration, the same amount was reverse transcribed into cDNA.

### 2.6. Western Blot

Treating cells with several comounds and then lysed using lysis buffer Ripa. Antibody information is as follows: BCL6 (14895S, CST), PARP (9532S, CST), CyclinB1 (12231S, CST), CyclinD1 (ab134175, CST), phospho-ERK (4370S, CST), Phospho-MEK (9121S, CST), AKT (4691S, CST), phospho-AKT (4060S, CST), c-MYC (CY5150, ABWAYS), GAPDH (AB0036, ABWAYS).

### 2.7. Xenograft Tumor Growth

The SKOV3 cell line was implanted into the back of 6/8-week female BALC/nude mice by subcutaneous injection. The number of cells inoculated per mouse was 1×10^7^ and the volume was 100 μL. Tumor size was measuredevery three days. As long as tumor size had risen to 100-150 mm^3,^ four groups was divided and treated by intraperitoneal injection of compounds. After drug treatment, all mice were euthanized, and tumors and organs were excised and further analyzed.

## 3. Results

### 3.1. BCL6 is overexpressed in ovarian cancer and which promotes ovarian cancer proliferation and metastasis

**Figure 1:**
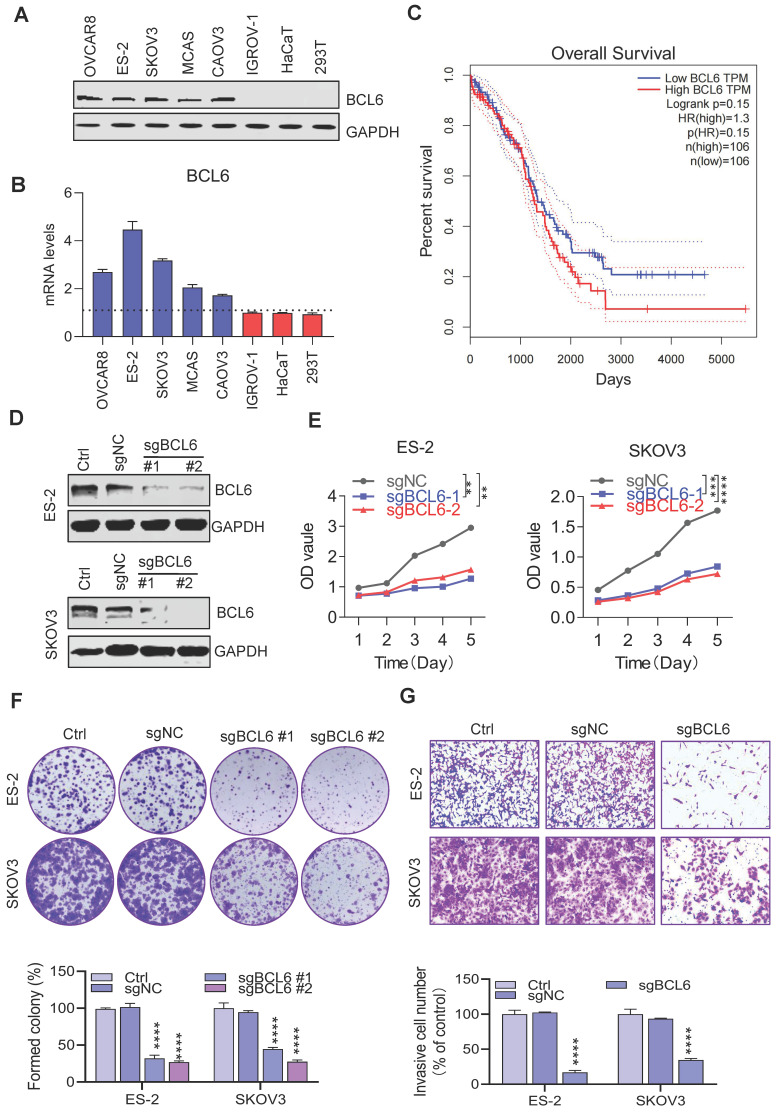
** BCL6 is overexpressed in ovarian cancer. (A)** Immunoblots of BCL6 in the ovarian cancer cell lines ES-2, SKOV3, OVCAR8, IGROV-1, CAOV3 and MCAS, as compared to the normal cells HaCaT and 293T.**(B)** RT-PCR data showing the transcript abundance of BCL6 in ovarian cancer cells lines ES-2, SKOV3, OVCAR8, IGROV-1, CAOV3 and MCAS, as compared to the normal cells HaCaT and 293T. BCL6 was calculated by normalization to GAPDH. **(C)** Using TCGA database to analyze the relationship between BCL6 expression and survival of ovarian cancer patients.** (D)** Generation of scrambled sgRNA (sgNC) and sgBCL6 cell lines from ES-2 and SKOV3 cells.** (E)** Cell viability readouts of sgNC and sgBCL6 cell lines derived from ES-2 and SKOV3 cells.** (F)** Clonogenic assay of sgNC and sgBCL6 cell lines derived from ES-2 and SKOV3 cells. A representative image of sgNC and sgBCL6 derived from ES-2 and SKOV3 cells colonies is shown. **(G)** Transwell assays of sgNC and sgBCL6 cell lines derived from ES-2 and SKOV3 cells, the number of invasive cells were evaluated after 24 h.

To elucidate the potential role of BCL6 in ovarian cancer, the transcript abundance and protein expression of BCL6 in mltiple human OV cells and normal cells were measured respectively. The protein level and the mRNA level of BCL6 in many OV cells was significantly higher than that in normal cells (Figure 1A-B). Our results were consistent with the CCLE database (Figure S1A), which further revealed that BCL6 has tumor type-specific expression in OV and potentially affects the proliferation and metastasis of OV. In addition, based on survival analysis conducted using the TCGA-OV database, it was found that there was no significant difference in prognosis between ovarian cancer patients with high and low expression levels of BCL6 (Figure 1C) . However, a separate study demonstrated that the expression of BCL6 was notably higher in OV tissues compared to the adjacent para-tumorous epithelium. Furthermore, there was a strong correlation between BCL6 expression and important clinical indicators such as FIGO staging, lymph node metastasis, and recurrence. Remarkably, OV patients with higher BCL6 expression levels exhibited significantly poorer disease-specific survival (DSS) and disease-free survival (DFS) outcomes^39^.

To validate whether BCL6 mediated the proliferation of OV, we used CRISPR/Cas9 technology to knockout the BCL6 gene in multiple strains of BCL6-overexpressed OV cells (Figure 1D). Notably, the cell proliferation abilities were tremendously reduced when BCL6 knockout in ES-2 and SKOV3 (Figure 1E). In addition, we also found that the cloning and migration capacity of cells were hindered after BCL6 knockout (Figure 1F-1G and Figure S1B-1D). Our results showed that BCL6 overexpression in IGROV-1 cells could enhance the proliferation, cloning and migration abilities (Figure S1E-1H). Taken together, our results further demonstrated that BCL6 was positively correlated with the migration, cloning and proliferation abilities of OV, suggesting that BCL6 may be an important potential target for the treatment of OV.

### 3.2. WK369 is a novel and potent of BCL6 small molecular inhibitor

**Figure 2:**
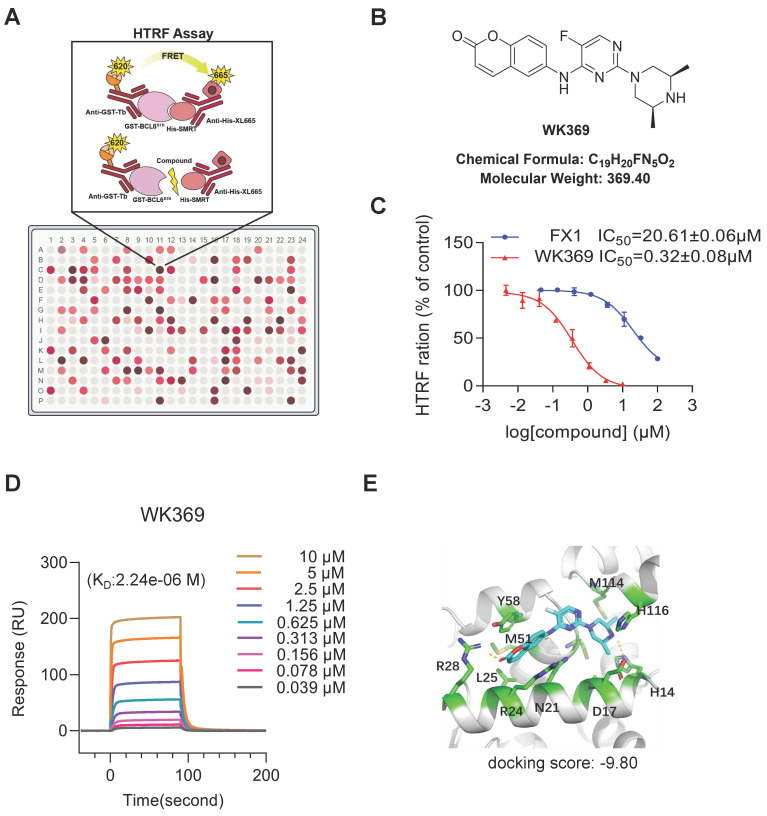
** WK369 was identified as a BCL6-BTB inhibitor. (A)** HTRF assay screening schematic.** (B)** Chemical structural of compound WK369.** (C)** HTRF assay measured the effect of WK369 or FX1 blocked the interaction between BCL6-BTB and SMRT.** (D)** SPR sensorgram of WK369 binding to BCL6-BTB, with WK369 concentrations and calculated K_D_ for binding shown.** (E)** Predicting the binding mode of WK369 with BCL6-BTB domain (PDB ID code: 5N1Z). The BCL6-BTB domain was shown as a cyan cartoon, and the inter residues were shown as green sticks.

Previous studies have shown that blocking the interaction of the BCL6-BTB domain with its corepressor has emerged as a promising strategy for designing BCL6 inhibitors^32^.To identify compounds capabilities to disturb the interaction between the BCL6-BTB domain with its transcriptional co-repressor SMRT for OV treatment, HTRF assay was established to screen the internal chemical library (Figure 2A). After screening, 60 compounds exhibited excellent binding affinities to BCL6-BTB domain (IC_50_ less than 2 μM), and WK369 showed the best affinities, which was approximately 120 times lower than that of FX1^37^ (Figure 2B-2C and Figure S2-S5). To further confirm whether WK369 could directly interact with BCL6-BTB domain, SPR assay were performed. As shown in Figure 2D, WK369 bound to BCL6-BTB domain with a K_D_ value of 2.24 μM. Molecular docking of WK369 was also performed on BCL6-BTB domain (PDB ID code: 5N1Z) to identify a putative binding site, and the result indicated that WK369 interacts with several important amino acid residues of BCL6^BTB^ including Tyr58, Met51, Arg28, Leu25 and His14. Among, Arg28 formed H-bond with the carbonyl oxygen of coumarin group at the left part of WK369; the H-bond was also formed between N-H of the piperazine ring of WK369 with His14; In addition, the benzene ring of coumarin group forms a Π-Π conjugation with Tyr58 (Figure 2E).

### 3.3. WK369 suppresses transcriptional inhibitory activity of BCL6 in vitro and in vivo

**Figure 3:**
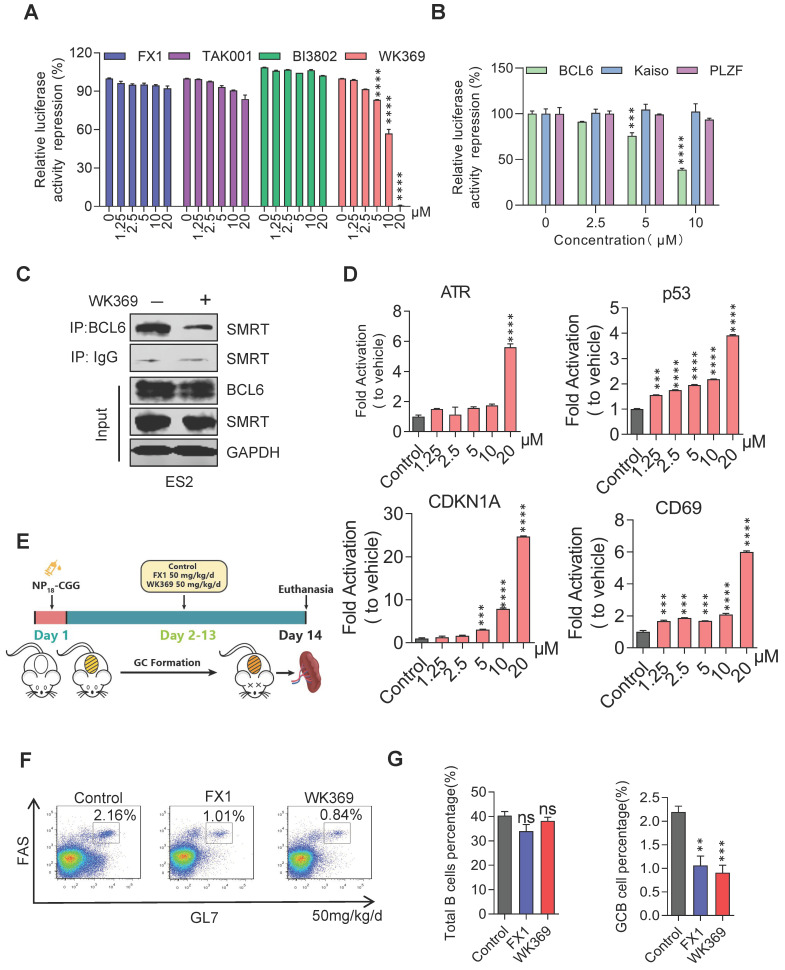
** WK369 suppresses transcriptional inhibitory activity of BCL6 *in vitro* and *in vivo*. (A)** WK369 inhibited BCL6-BTB mediated transcriptional repression in luciferase reporter assays.** (B)** Reporter assays were performed to test the activities of WK369 with different BTB-related proteins (BCL6, Kaiso and PLZF). **(C)** After 24 h treatment with 5 μM WK369, ovarian cancer cells lysates were immunoprecipitated with BCL6 overnight. The precipitates were washed, suspended in non-reducing sample buffer, and boiled for 10 min. The indicated antibodies were used for western blot assays. **(D)** Farage cells exposed to 24 h treatment with different concentrations of WK369 were evaluated for BCL6-target genes (ATR, p53, CDKN1A and CD69) expression by RT-PCR. **(E)** C57/BL6 mice were immunized with NP_18_-CGG and by intraperitoneal administration WK369 at a dosage of 50 mg/kg/d for 12 days to further detect whether WK369 can inhibit the transcriptional inhibitory function of BCL6 *in vivo.* **(F)** Flow cytometry detection of splenic GC B cells (B220+GL7+FAS+) percentage. **(G)** Statistical graph of the total number of B cells or the percentage of GC-B cells in the mouse spleen. *P < 0.05, **P < 0.01, ***P < 0.001, ****P < 0.0001, n.s. not significant by log-rank (Mantel-Cox) test.

To confirm whether WK369 can inhibit the transcription inhibitory activity of BCL6-BTB, a luciferase reporter assay was constructed by transferring the GAL4-TK-luc, Renilla, GAL4-DBD plasmid or GAL4-TK-luc, Renilla, GAL4-DBD-BCL6 plasmid into the 293T cells. After treatment of WK369 for 24 h, we found that, however the positive control groups (FX1^37^, TAK001^35^ and BI3802^33^) had little inhibitory effects even at 20 μM (Figure 3A). WK369 inhibited the transcriptional repressor activity of BCL6-BTB in a dose dependent manner, but without any effect on the other BTB zinc finger repressors (Kaiso and PLZF) (Figure 3B). Co-IP experiments show that the interaction between BCL6 with SMRT was significant inhibited (Figure 3C). In addition, to verify whether WK369 can reactivate the BCL6-target genes, Farage cell was exposed to WK369. Consistent with the results of luciferase activities, RT-qPCR showed that WK369 could significantly reactivate multiple BCL6-target genes including ATR, CD69, p53 and CDKN1A (Figure 3D).

BCL6 plays a significant role in the formation of germinal centers (GCs), the formation of GCs and affinity maturation of immunoglobulins were disrupted in the mice with mutated BCL6-BTB domain^40^. Therefore, to further examine the ability of WK369 to inhibit activity of BCL6 in vivo, we immunized C57/BL6 mice with NP18-CGG and further analyzed (Figure 3E). Flow cytometry results analysis showed the overall B cells in the WK369 administration group had no effect, but the proportion of GC B cells was reduced and lower of FX1 (Figure 3F-3G). As above mention that the mice with mutation of BCL6-BTB also result in impairing immunoglobulin affinity maturation^40^, thus we next tested the NP-specific antibody content in serum by ELISA. As expected, the high-affinity IgG1 (NP5 IgG1) and total IgG1 (NP23 IgG1) secreted were also remarkably decreased after treatment of WK369 (Figure S6). Taken together, these findings indicated that WK369 could tremendously suppressed the transcriptional inhibitory activity of BCL6-BTB *in vitro* and *in vivo* by directly binding to the BCL6-BTB domain, and WK369 was a potential BCL6 inhibitor.

### 3.4. WK369 inhibits ovarian cancer cell growth, migration and induces apoptosis

**Figure 4:**
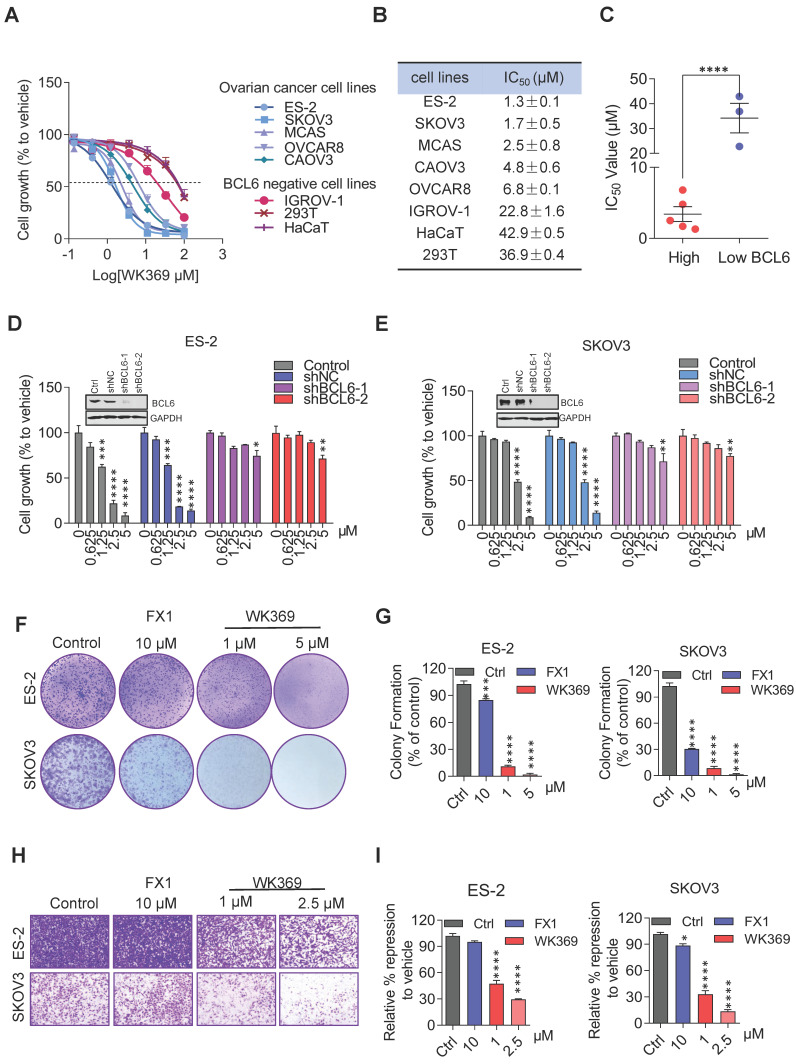
** WK369 inhibits ovarian cancer cell proliferation. (A)** MTS assays from 48 h WK369 treatment assessed the viability of multiple ovarian cancer cell lines, as compared to normal human cell lines.** (B)** Antiproliferative activities of WK369 on different ovarian cancer cell lines and cytotoxicities on normal cell lines. **(C)** Statistical graph of activity difference of WK369 between high and low expression of BCL6 in different cell lines (right). **(D-E)** Cell viability assay of ES-2 and SKOV3 cells expressing BCL6 specific shRNA (shBCL6-1 and shBCL6-2) or Scramble shRNA (shScr) with indicated concentrations of WK369 treatment for 72 h. **(F-G)** Clonogenic assay of ES-2 and SKOV3 cells treated with the indicated concentrations of WK369 or FX1 for 7 days. A representative image of ES-2 and SKOV3 colonies is shown.** (H-I)** Anti-migration effects of WK369 or FX1 on ES-2 and SKOV3 after 24 h of treatment through transwell cell invasion assays. *P < 0.05, **P < 0.01, ***P < 0.001, ****P < 0.0001, n.s. not significant by log-rank (Mantel-Cox) test.

**Figure 5:**
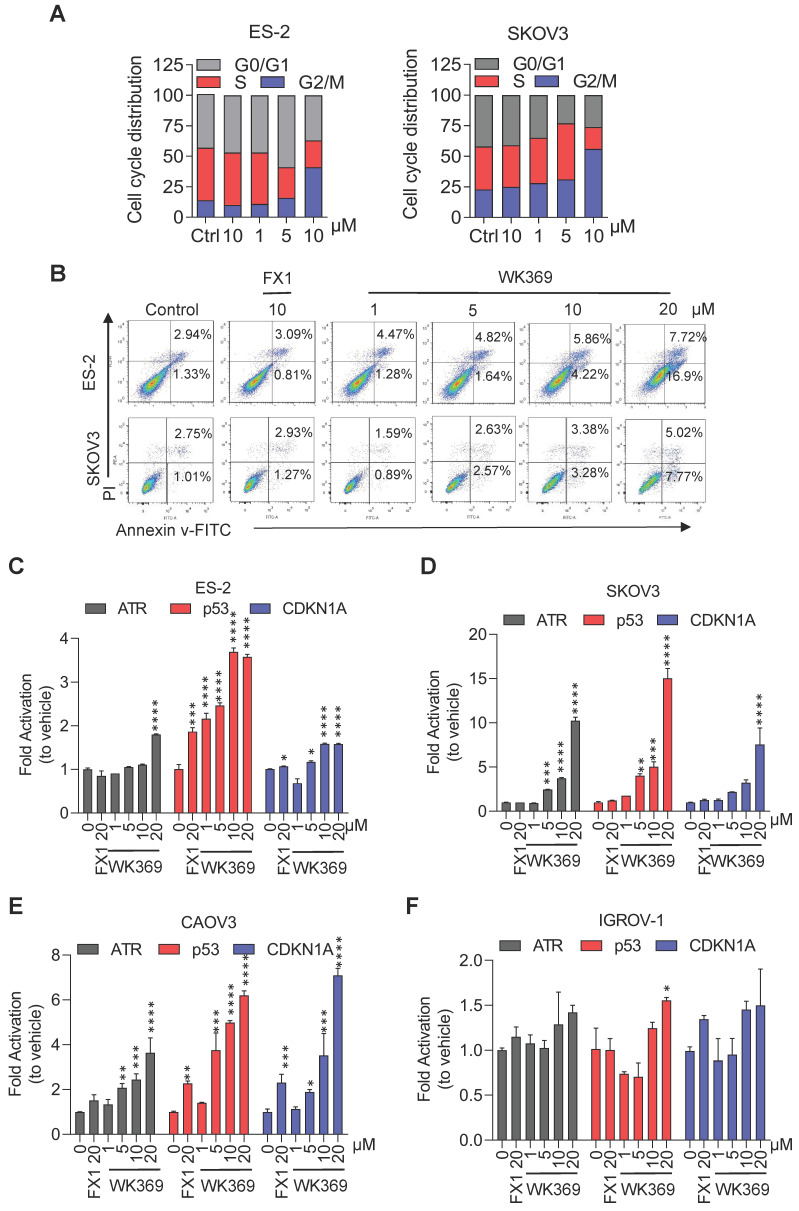
** WK369 induces ovarian cancer cell apoptosis and reactivates of BCL6 target genes. (A)** Flow cytometry analysis showing the effects of 24h indicated concentrations of WK369 or FX1 treatment of ES-2 or SKOV3 cells on cell cycle arrest and accumulation in the S phase.** (B)** Apoptosis effects of ES-2 or SKOV3 cells after treatment with FX1 and WK369 for 48 h. **(C-F)** Changes in BCL6 target genes transcript abundance after 24h WK369 treatment with the indicated concentrations in ES-2, SKOV3, HO8910PM and IGROV-1 cell lines. *P < 0.05, **P < 0.01, ***P < 0.001, ****P < 0.0001, n.s. not significant by log-rank (Mantel-Cox) test.

In anti-tumor proliferation experiments, WK369 inhibited cell proliferation in a variety of OV cells that highly expressed BCL6, but had little effect on normal cells that were independent of BCL6 (Figure 4A-4C). Meanwhile, WK369 showed better anti-ovarian cancer activity and anti-DLBCL cancer cell activity than BCL6 inhibitors FX1 and WK500B (Figure S7). To further validate the BCL6 is indispensable for WK369 inhibitory effects, ES-2 and SKOV3 cells with lentivirus expressing shBCL6 or shNC were treated with WK369. Notably, WK369 had little effect on shBCL6 cells and inhibited the proliferation of shNC cells, indicating that WK369 inhibited the proliferation of OV mainly by targeting BCL6 (Figure 4D-4E). Furthermore, WK369 significantly inhibited OV cells clonal formation (Figure 4F-4G and Figure S8A-S8B) and also prevented the invasion and migration ability of OV cells (Figure 4H-I and Figure S8C-S8D), which was more potently than that of FX1. As analyzed by flow cytometry, OV cells treated with WK369 induced cell cycle arrest, with accumulation in the S phase (Figure 5A). Moreover, we also found that WK369 significantly induced OV cells apoptosis (Figure 5B).

Considering that BCL6 mediates tumorigenesis by inhibiting BCL6-target genes, thus we speculated that the mechanism for efficiently killing OV cells by WK369 may be through reactivating these target genes. To verify our conjecture, ES-2, SKOV3, CAOV3 (overexpression of BCL6) and IGROV-1 cells (low-expression of BCL6, as negative control) were exposed to WK369. As expected, the BCL6-target genes ATR, p53 and CDKN1A were significantly up-regulated in ES-2, SKOV3 and CAOV3, while has little effect in IGROV-1 (Figure 5C-5F). Subsequent studies also found that the expression of CyclinB1 and CyclinD1 were downregulated and the apoptosis related protein PARP was activated when treatment of WK369 (Figure S8E-S8F). The immunofluorescence assay found that DNA damage-related marker γH2AX was overexpression after treated with WK369 (Figure S8G).

### 3.5. WK369 prevents ovarian cancer growth in vivo

**Figure 6:**
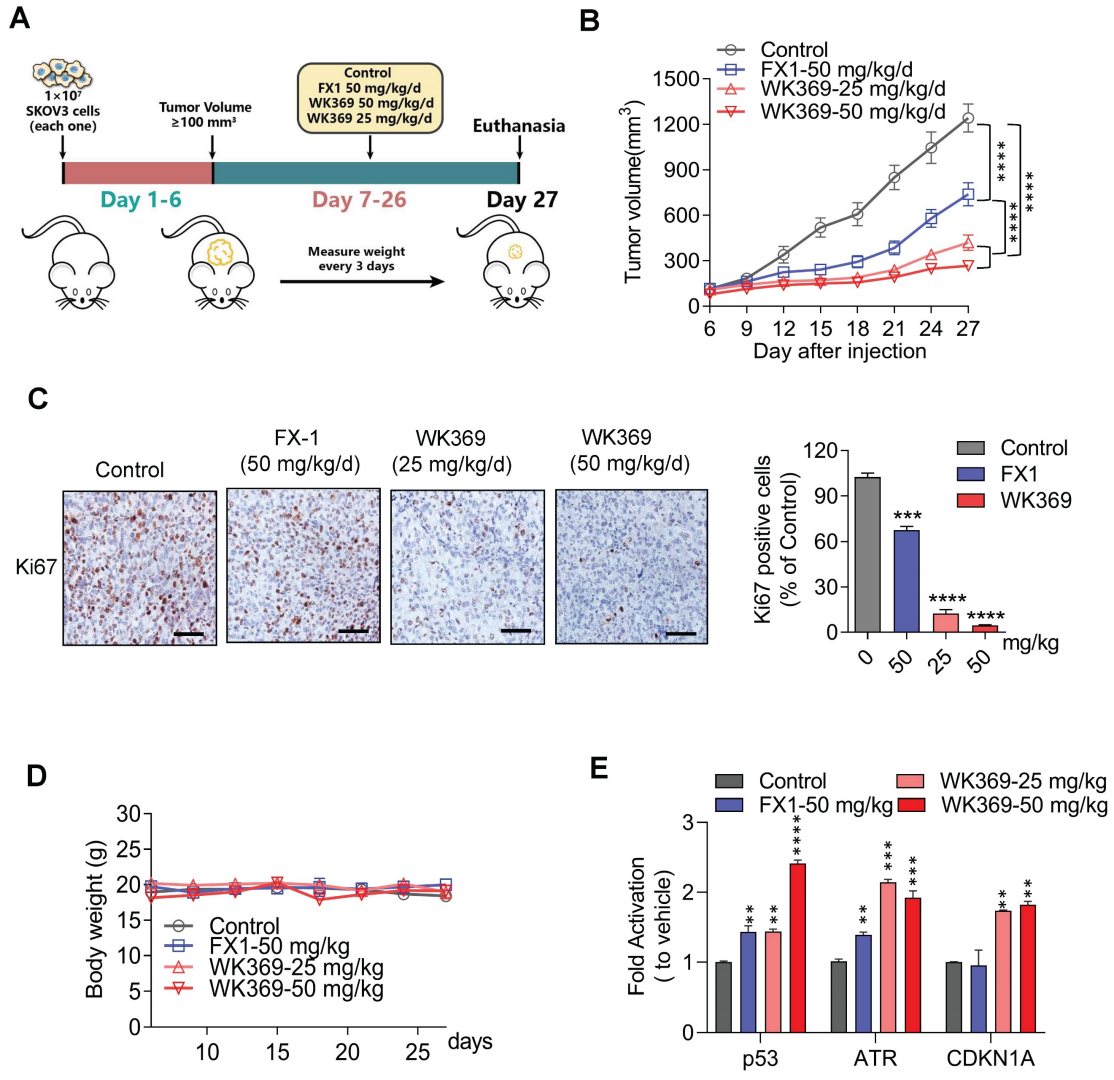
** Antitumor effects of WK369 on SKOV3 xenograft mouse models. (A)** SKOV3 xenograft mice were administered WK369 (25 mg/kg/d) and WK369 (50 mg/kg/d), with FX1 (50 mg/kg/d) as a control (n = 7 mice per group). **(B)** Tumour volumes (mm^3^) evaluated once every 3 days for a total of 21 days.** (C)** Images of IHC staining of Ki67 are shown. Scale bars, 50 μm. **(D)** Body weights changes of each group on day 21. **(E)** mRNA expression of the BCL6 target genes from tumors was measured by RT-PCR assays. *P < 0.05, **P < 0.01, ***P < 0.001, ****P < 0.0001, n.s. not significant by log-rank (Mantel-Cox) test.

We next constructed SKOV3 xenograft mouse model to assess the anti-tumor activity of WK369 *in vivo* (Figure 6A). The mices were averagely divided into four groups: vehicle group, positive control group (FX1-50 mg/kg), WK369-25 mg/kg and WK369-50 mg/kg. As expected, WK369 could significantly suppress tumor growth with dose-dependent compared with that in the vehicle, and it displayed a markedly potent effect than that of the FX1 group (Figure 6B and S9A). In addition, IHC staining of Ki67, a proliferation marker, was dramatically decreased after WK369 administration compared to controls (Figure 6C), these data underline that WK369 achieves superior anti-ovarian cancer effects *in vivo*. At the same time, H&E staining revealed that there was no significant toxicity after WK369 treatment (Figure S9B). In addition, no obvious weight changes of mice in both WK369 treatment groups and blank control group were observed (Figure 6D). Remarkably, WK369 significantly induced the upregulation of BCL6 target genes *in vivo* (Figure 6E), which is consistent with our *in vitro* data observed in ovarian cancer cells treated with WK369, confirming the BCL6 inhibitory effect of WK369 *in vivo*. Furthermore, we check the change of the composition and function of immune cells in mice upon WK369 treatment and found that there was no significant change overall immune cells and immune B cells in mice (Figure S9C-E).

### 3.6. WK369 suppress ovarian cancer intra-abdominal metastasis in vivo

**Figure 7:**
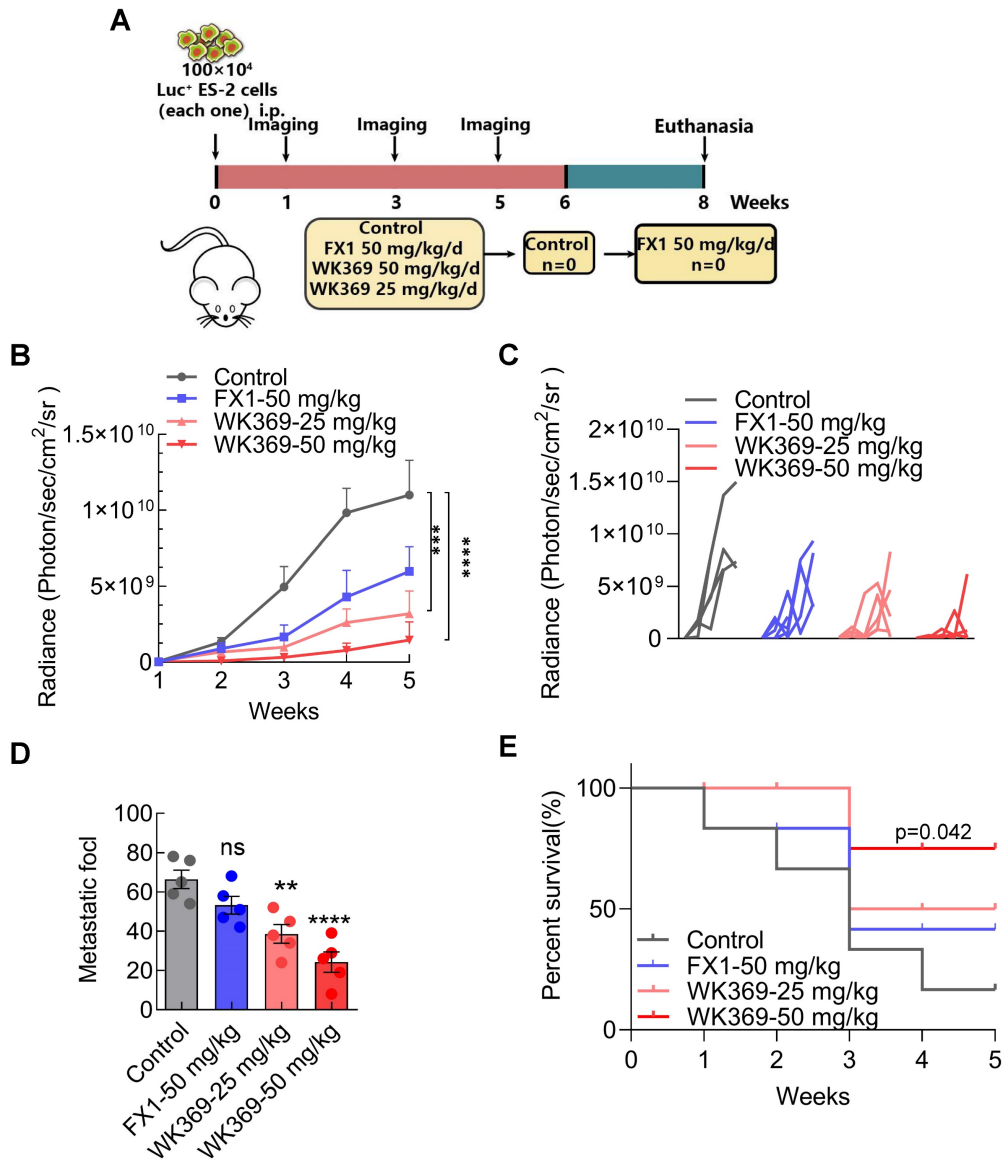
** A mouse model of ovarian cancer metastasis showed that WK369 had powerful anti-metastatic properties, which prolonged the survival of tumor-bearing mice. (A)** ES-2 cells expressing luciferase were intrasplenically injected into BALB/c-nude mice, and tumor growth was assessed by IVIS and quantified by bioluminescence imaging.** (B-C)** Data represent the mean ± SD; ***P < 0.001, ****P < 0.0001, n.s. not significant by one-way ANOVA followed by multiple-comparison tests. **(D)** After 5 weeks, mice were sacrificed and organs were imaged. **(E)** Overall survival rate of mice in the different treatment groups (n = 5 mice per group).

Our finding suggested that WK369 could significantly inhibit ovarian cancer migration *in vitro*, which prompting us to further test whether WK369 can suppress ovarian cancer metastasis *in vivo*. It was assessed using an ovarian cancer intra-abdominal metastasis model. ES-2 cells expressing luci were injected into the abdominal cavity of BLAB/c female nude mice and the tumor metastasis was monitored weekly. Based on their bioluminescence signals, the mice were randomly divided into 4 groups: control, positive control group FX1-50 mg/kg, WK369-25 mg/kg and WK369-50 mg/kg (Figure 7A). We used a small animal *in vivo* optical imaging system (IVIS) to monitor the tumor metastasis signal in real-time every week (Figure S10A). Exciting is that either dose of WK369 significantly inhibited metastasis of ovarian cancer, and their effects were more potent than that of FX1 treatment (Figure 7B-7D). Sacrifice mice at the end of the experiment, and the liver, spleen, intestine, kidney and ovary were immediately removed for taking pictures and counting tumor metastasis nodules in each group. The results showed that WK369 reduced the number of tumor nodules in various organs compared with the control group, and the effects were more potent than that of the FX1 group (Figure S10B-S10C). Given that WK369 could suppress ovarian cancer metastasis, we speculated that WK369 would prolong the survival period of mice. To verify this hypothesis, we tested the survival rate of mice during the administration period. To our surprise, WK369 significantly prolong the survival of tumor-bearing mice, and its effect was more potent than that of FX1 (Figure 7E). Collectively, our data indicated that WK369 could effectively inhibit the intraperitoneal metastasis of ovarian cancer in the preclinical mouse model and prolong the survival rates of tumor-bearing mice.

### 3.7. Therapeutic targeting of BCL6 sensitizes ovarian cancer to chemotherapy

**Figure 8:**
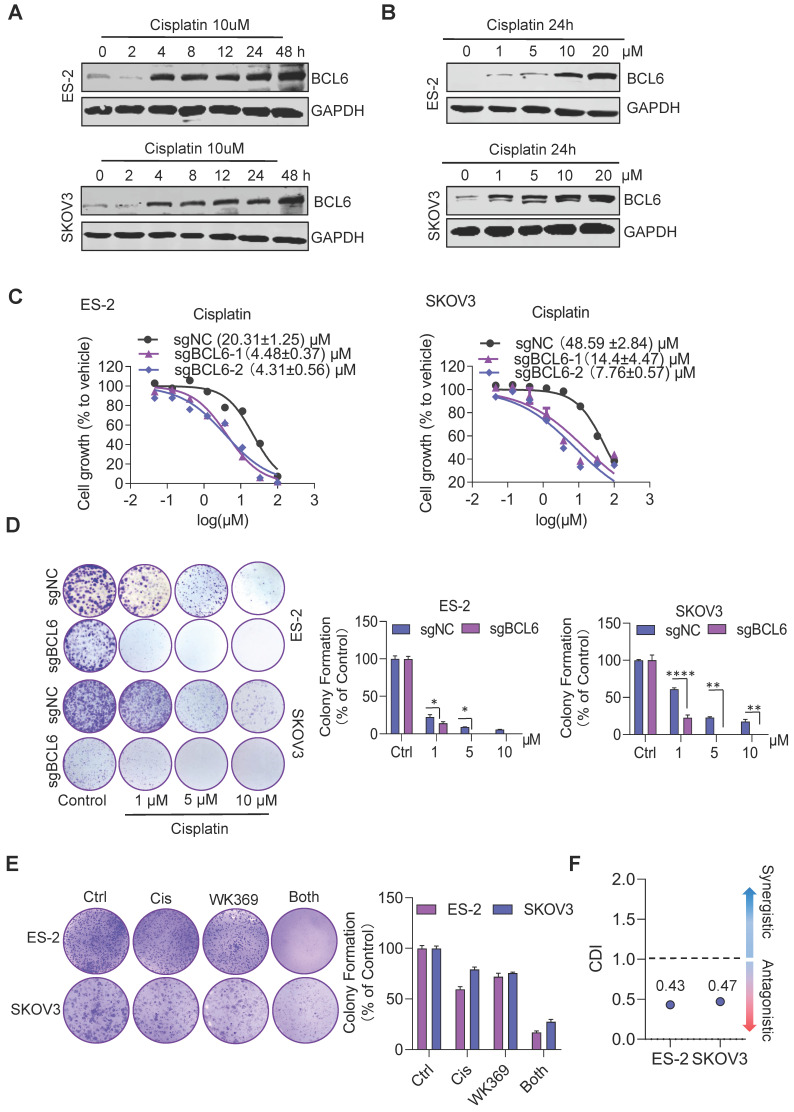
** WK369 restores the sensitivity of drug-resistant ovarian cancer cell lines to cisplatin. (A)** Expression levels of BCL6 were detected by western blot following treatment of cisplatin (10 μM) at different times in ES-2 and SKOV3 cells. **(B)** Expression levels of BCL6 were detected by western blot following treatment of different concentrations of cisplatin in ES-2 and SKOV3 cells for 24 h.** (C)** The scrambled sgRNA or BCL6 sgRNA1# and 2# was transfected in ES-2 and SKOV3 cells. After 48 h transfection, transfected cells were seeded in 96-well plates and were treated with cisplatin for 48 h, and the viability of cells was determined using the MTS assay. **(D)** The same series of ovarian cancer cells as (**C**) were subjected to colony formation assays were treated with cisplatin after 48 h. Colonies were photographed, enumerated, and analyzed after 7 days.** (E)** Colony formation after treatment with cisplatin, WK369 and combination for 12 days. The coefficient of drug interaction (CDI) was calculated using the mean% survival and colony formation of a single representative experiment with three determinations. CDI < 1 indicated synergism, CDI < 0.7 significant synergism, CDI = 1 additivity, CDI > 1 antagonism. Source data are provided as a source data file.

Previous studies have shown that BCL6 is related to cisplatin resistance in ovarian cancer^29^. Consistent with this result, we also found that cisplatin could induce consistent upregulation of BCL6 (Figure 8A-8B), and knockout BCL6 could enhance the sensitivity of drug-resistant OV cell lines to cisplatin (Figure 8C-8D). To quantify drug interactions between WK369 and cisplatin, two softwares were used: CompuSyn and SynergyFinder. The cell viability of SKOV3 and ES-2 was detected after WK369 combined with cisplatin, and it was found that the anti-tumor activity of the combination treatment group was significantly higher than that of the single-drug group, and the CI values were all below 1, indicating a synergistic effect (Figure S11A-B). Analysing the synergy plots of WK369 and cisplatin, it was found that the drug interaction in this combination was synergistic by HAS models, SKOV3 with synergy scores of 11.877 and ES-2 with synergy scores of 14.119 (Figure S11C-F). As a result, co-treatment with WK369 and cisplatin significantly inhibited the colony formation in SKOV3 and ES-2, which was more potent than cisplatin administering alone (Figure 8E). Collectively these data suggested that WK369 may become a new regimen for ovarian cancer chemotherapy resistance.

### 3.8. WK369 inhibits AKT and MEK/ERK signaling pathway by targeting BCL6

Previous studies found that 30%-40% of ovarian cancers have abnormal MEK/ERK signaling pathway, which promotes proliferation and survival of ovarian cancer. In addition, epithelial low differentiated ovarian cancer is often accompanied by mutation and phosphorylation of AKT, which is closely related to tumor invasion and metastasis^41^,^42^. Knockout BCL6 can inhibit the phosphorylation and activation of AKT and MEK-ERK, indicating that BCL6 may regulate the cascade of AKT and MEK-ERK^43^. Therefore, we speculated that WK369 suppressed ovarian cancer growth and metastasis may inhibit the activation of AKT and MEK/ERK signaling pathway. To verify our hypothesis, ES-2 and SKOV3 with high expression of BCL6 were treated with WK369. After 24 h, WK369 can significantly inhibit the phosphorylation of AKT and MER/ERK with concentration-dependent (Figure S12A-S12B). Taken together, our data suggested that WK369 inhibited the activation of AKT and MEK-ERK by blocking the function of BCL6 to inhibit the proliferation, survival and metastasis of OV cells, and promote the occurrence of cycle arrest and apoptosis.

Since WK369 has positive biological activity both in vitro and in vivo, we further conducted preclinical pharmacokinetic characterization studies. The results show that WK369 has acceptable metabolic stability in vivo and is widely distributed in tissues. More importantly, WK369 has excellent oral bioavailability (F = 72.36%), suggesting that WK369 can be further developed as an oral anticancer drug (Figure S13). In addition, WK369 did not cause death in mice even at a dose of 1000 mg/kg.

## 4. Discussion

OV is known as the “silent killer” due to its difficulty in early diagnosis and the vast majority of patients have distal metastases when diagnosed. The majority of patients get good feedback after initial surgery and platinum-based chemotherapy, however about 75% of patients will relapse and develop drug resistance^8^,^44^. BCL6 is a newly discovered drug target in recent years. Emerging evidence has indicated that BCL6 promotes malignant transformation, and targeted therapy of BCL6 has been extensively studied in multiple types of tumors^26^,^27^,^37^. However, BCL6 targeted therapy in ovarian cancer is rarely reported. In this study, we demonstrated that BCL6 was overexpressed in OV cells compared to normal cells and knockout BCL6 could significantly inhibit the proliferation of OV cells, which was consistent with the previous report that BCL6 was commonly expressed in OV and associated with poor prognosis^28^. To develop BCL6 inhibitor for treatment of ovarian cancer, we established HTRF and BCL6 luciferase reporter screening system, and finally WK369 was identified as a novel BCL6 inhibitor, which could disturb the interaction between BCL6 and SMRT at low concentrations and significantly re-activate the BCL6 target genes *in vitro*. More importantly, it also could sharply inhibit BCL6 biology function *in vivo*, and its effect was better than that of FX1. Subsequently research showed that WK369 was more potency in suppressing OV growth *in vitro* and *in vivo* than FX1.

Metastasis is one of the major problems that hinder the long-term survival of OV patients. A previous study revealed that BCL6 promoted OV cells invasion and migration through inducing the invasion-promoting genes such as N-Cadherin, MMP2 and MMP9^28^. Our studies also indicated that knockout BCL6 inhibited OV cells migration. Therefore, we evaluated the effects of WK369 on the migration of OV and found that WK369 could significantly suppress the OV migration *in vitro* and *in vivo*, and prolong the OS. Intrinsic or acquired cisplatin resistance is another therapeutic problem for ovarian cancer^3^,^45^. Here, we also found that BCL6 was upregulated in a time- and concentration- dependent manner upon treatment of cisplatin, and knockout BCL6 could increase their sensitivity to cisplatin. WK369 improved the tumor response and enhanced the sensitivity of OV cisplatin-resistant cell lines to cisplatin, and down-regulated BCL6 expression. These results indicate that WK369 can be used as a lead compound to treat ovarian cancer metastasis and overcome cisplatin resistance.

As an oncogene, BCL6 promotes the malignant transformation by inhibiting the expression of target genes including DNA damage sensing, ATR^21^, p53^46^, and cell cycle arrest gene CDKN1A^23^,^24^. Therefore, we speculated that BCL6 inhibition through WK369 activating the expression of these genes to suppress ovarian cancer growth and metastasis. Just as we thought, p53, ATR and CDKN1A were significantly activated after treatment of WK369 in OV cells. Besides, the phosphorylation of AKT and MEK-ERK was significantly suppressed after treatment of WK369 in ES-2 and SKOV3 cells. Taken together, our data indicated that WK369 exerts potent anti-tumor activities may be through reactivating p53, ATR and CDKN1A, inhibiting AKT and MEK/ERK signaling pathways.

We not only validated the efficacy of the identified BCL6 inhibitor WK369 as a monotherapy for ovarian cancer, but also provided a potential therapeutic option for the use of targeted agents in adjuvant chemotherapy. In summary, we identified WK369 as a novel BCL6 small molecular inhibitor, which could significantly inhibit the growth and migration of OV *in vitro* and *in vivo* without toxicity and prolonged the survival of tumor-bearing mice. Furthermore, WK369 could suppress OV cells with acquired resistance to cisplatin, and enhanced the sensitivity of OV cisplatin-resistant cell lines to cisplatin. Therefore, WK369 may provide a new combination strategy with cisplatin for the treatment of ovarian cancer.

## Supplementary Material

Supplementary methods and figures.

## References

[1] Siegel RL, Miller KD, Fuchs HE, Jemal A (2022). Cancer statistics, 2022. *CA: a cancer journal for clinicians*.

[2] Torre LA, Trabert B, DeSantis CE, Miller KD, Samimi G, Runowicz CD (2018). Ovarian cancer statistics, 2018. *CA: a cancer journal for clinicians*.

[3] Vaughan S, Coward JI, Bast RC Jr, Berchuck A, Berek JS, Brenton JD (2011). Rethinking ovarian cancer: recommendations for improving outcomes. *Nat Rev Cancer*.

[4] Xiao Y, Yu Y, Gao D, Jin W, Jiang P, Li Y (2019). Inhibition of CDC25B With WG-391D Impedes the Tumorigenesis of Ovarian Cancer. *Front Oncol*.

[5] Ebell MH, Culp MB, Radke TJ (2016). A Systematic Review of Symptoms for the Diagnosis of Ovarian Cancer. *Am J Prev Med*.

[6] Li B, Tong T, Ren N, Rankin GO, Rojanasakul Y, Tu Y (2021). Theasaponin E1 Inhibits Platinum-Resistant Ovarian Cancer Cells through Activating Apoptosis and Suppressing Angiogenesis. *Molecules*.

[7] Gibson BA, Kraus WL (2012). New insights into the molecular and cellular functions of poly(ADP-ribose) and PARPs. *Nat Rev Mol Cell Biol*.

[8] Cortesi L, Toss A, Cucinotto I (2018). PARP Inhibitors for the Treatment of Ovarian Cancer. *Curr Cancer Drug Targets*.

[9] Matulonis UA, Penson RT, Domchek SM, Kaufman B, Shapira-Frommer R, Audeh MW (2016). Olaparib monotherapy in patients with advanced relapsed ovarian cancer and a germline BRCA1/2 mutation: a multistudy analysis of response rates and safety. *Ann Oncol*.

[10] Scott LJ (2017). Niraparib: First Global Approval. *Drugs*.

[11] Syed YY (2017). Rucaparib: First Global Approval. *Drugs*.

[12] Bennet N (2012). No survival benefit of olaparib in ovarian cancer patients. *The Lancet Oncology*.

[13] Chappell NP, Miller CR, Fielden AD, Barnett JC (2016). Is FDA-Approved Bevacizumab Cost-Effective When Included in the Treatment of Platinum-Resistant Recurrent Ovarian Cancer?. *J Oncol Pract*.

[14] Pujade-Lauraine E, Hilpert F, Weber B, Reuss A, Poveda A, Kristensen G (2014). Bevacizumab combined with chemotherapy for platinum-resistant recurrent ovarian cancer: The AURELIA open-label randomized phase III trial. *J Clin Oncol*.

[15] Burger RA, Brady MF, Bookman MA, Fleming GF, Monk BJ, Huang H (2011). Incorporation of bevacizumab in the primary treatment of ovarian cancer. *N Engl J Med*.

[16] Perren TJ, Swart AM, Pfisterer J, Ledermann JA, Pujade-Lauraine E, Kristensen G (2011). A phase 3 trial of bevacizumab in ovarian cancer. *N Engl J Med*.

[17] Topalian SL, Drake CG, Pardoll DM (2015). Immune checkpoint blockade: a common denominator approach to cancer therapy. *Cancer Cell*.

[18] Patel SA, Minn AJ (2018). Combination Cancer Therapy with Immune Checkpoint Blockade: Mechanisms and Strategies. *Immunity*.

[19] Brahmer JR, Tykodi SS, Chow LQ, Hwu WJ, Topalian SL, Hwu P (2012). Safety and activity of anti-PD-L1 antibody in patients with advanced cancer. *N Engl J Med*.

[20] Gaillard SL, Secord AA, Monk B (2016). The role of immune checkpoint inhibition in the treatment of ovarian cancer. *Gynecol Oncol Res Pract*.

[21] Ranuncolo SM, Polo JM, Dierov J, Singer M, Kuo T, Greally J (2007). Bcl-6 mediates the germinal center B cell phenotype and lymphomagenesis through transcriptional repression of the DNA-damage sensor ATR. *Nat Immunol*.

[22] Cheng H, Linhares BM, Yu W, Cardenas MG, Ai Y, Jiang W (2018). Identification of Thiourea-Based Inhibitors of the B-Cell Lymphoma 6 BTB Domain via NMR-Based Fragment Screening and Computer-Aided Drug Design. *J Med Chem*.

[23] Phan RT, Saito M, Basso K, Niu H, Dalla-Favera R (2005). BCL6 interacts with the transcription factor Miz-1 to suppress the cyclin-dependent kinase inhibitor p21 and cell cycle arrest in germinal center B cells. *Nat Immunol*.

[24] Ranuncolo SM, Polo JM, Melnick A (2008). BCL6 represses CHEK1 and suppresses DNA damage pathways in normal and malignant B-cells. *Blood Cells Mol Dis*.

[25] Leeman-Neill RJ, Bhagat G (2018). BCL6 as a therapeutic target for lymphoma. *Expert opinion on therapeutic targets*.

[26] Xu L, Chen Y, Dutra-Clarke M, Mayakonda A, Hazawa M, Savinoff SE (2017). BCL6 promotes glioma and serves as a therapeutic target. *Proc Natl Acad Sci U S A*.

[27] Walker SR, Liu S, Xiang M, Nicolais M, Hatzi K, Giannopoulou E (2015). The transcriptional modulator BCL6 as a molecular target for breast cancer therapy. *Oncogene*.

[28] Wang YQ, Xu MD, Weng WW, Wei P, Yang YS, Du X (2015). BCL6 is a negative prognostic factor and exhibits pro-oncogenic activity in ovarian cancer. *Am J Cancer Res*.

[29] Shen J, Hong L, Chen L (2020). Ubiquitin-specific protease 14 regulates ovarian cancer cisplatin-resistance by stabilizing BCL6 oncoprotein. *Biochem Biophys Res Commun*.

[30] Bellenie BR, Cheung KJ, Varela A, Pierrat OA, Collie GW, Box GM (2020). Achieving In Vivo Target Depletion through the Discovery and Optimization of Benzimidazolone BCL6 Degraders. *J Med Chem*.

[31] McCoull W, Abrams RD, Anderson E, Blades K, Barton P, Box M (2017). Discovery of Pyrazolo[1,5-a]pyrimidine B-Cell Lymphoma 6 (BCL6) Binders and Optimization to High Affinity Macrocyclic Inhibitors. *J Med Chem*.

[32] McCoull W, Cheung T, Anderson E, Barton P, Burgess J, Byth K (2018). Development of a Novel B-Cell Lymphoma 6 (BCL6) PROTAC To Provide Insight into Small Molecule Targeting of BCL6. *ACS Chem Biol*.

[33] Kerres N, Steurer S, Schlager S, Bader G, Berger H, Caligiuri M (2017). Chemically Induced Degradation of the Oncogenic Transcription Factor BCL6. *Cell reports*.

[34] Sameshima T, Yamamoto T, Sano O, Sogabe S, Igaki S, Sakamoto K (2018). Discovery of an Irreversible and Cell-Active BCL6 Inhibitor Selectively Targeting Cys53 Located at the Protein-Protein Interaction Interface. *Biochemistry*.

[35] Kamada Y, Sakai N, Sogabe S, Ida K, Oki H, Sakamoto K (2017). Discovery of a B-Cell Lymphoma 6 Protein-Protein Interaction Inhibitor by a Biophysics-Driven Fragment-Based Approach. *Journal of medicinal chemistry*.

[36] Cerchietti LC, Ghetu AF, Zhu X, Da Silva GF, Zhong S, Matthews M (2010). A small-molecule inhibitor of BCL6 kills DLBCL cells in vitro and in vivo. *Cancer cell*.

[37] Cardenas MG, Yu W, Beguelin W, Teater MR, Geng H, Goldstein RL (2016). Rationally designed BCL6 inhibitors target activated B cell diffuse large B cell lymphoma. *The Journal of clinical investigation*.

[38] Xing Y, Guo W, Wu M, Xie J, Huang D, Hu P (2022). An orally available small molecule BCL6 inhibitor effectively suppresses diffuse large B cell lymphoma cells growth in vitro and in vivo. *Cancer letters*.

[39] Wang YQ XM, Weng WW, Wei P, Yang YS, Du X (2014). BCL6 is a negative prognostic factor and exhibits pro-oncogenic activity in ovarian cancer. Am J Cancer Res.

[40] Huang C, Hatzi K, Melnick A (2013). Lineage-specific functions of Bcl-6 in immunity and inflammation are mediated by distinct biochemical mechanisms. *Nature immunology*.

[41] Linnerth-Petrik NM, Santry LA, Moorehead R, Jucker M, Wootton SK, Petrik J (2016). Akt isoform specific effects in ovarian cancer progression. *Oncotarget*.

[42] Mabuchi S, Kuroda H, Takahashi R, Sasano T (2015). The PI3K/AKT/mTOR pathway as a therapeutic target in ovarian cancer. *Gynecol Oncol*.

[43] Song W, Wang Z, Kan P, Ma Z, Wang Y, Wu Q (2018). Knockdown of BCL6 Inhibited Malignant Phenotype and Enhanced Sensitivity of Glioblastoma Cells to TMZ through AKT Pathway. *Biomed Res Int*.

[44] Zhong L, Pan Y, Shen J (2021). FBXW7 inhibits invasion, migration and angiogenesis in ovarian cancer cells by suppressing VEGF expression through inactivation of beta-catenin signaling. *Exp Ther Med*.

[45] Wei X, Shi J, Lin Q, Ma X, Pang Y, Mao H (2021). Targeting ACLY Attenuates Tumor Growth and Acquired Cisplatin Resistance in Ovarian Cancer by Inhibiting the PI3K-AKT Pathway and Activating the AMPK-ROS Pathway. *Front Oncol*.

[46] Phan RT, Dalla-Favera R (2004). The BCL6 proto-oncogene suppresses p53 expression in germinal-centre B cells. *Nature*.

