# Supplementary material for: Selectively targeting BCL6 using a small molecule inhibitor is a potential therapeutic strategy for ovarian cancer
Source: Int J Biol Sci. 2024 Jan 1;20(2):486–501. doi: 10.7150/ijbs.86303 (PMC10758095; doi:10.7150/ijbs.86303)
Supplement: Supplementary file 1 — Supplementary methods and figures. [file ijbsv20p0486s1.pdf]

Supplemental Materials for

Selectively targeting BCL6 using a small molecule inhibitor is a potential therapeutic strategy for ovarian cancer

Supplementary methods:

Synthesis and characterization of WK369. To a solution of compounds 6-aminocoumarin hydrochloridc (197.62 mg, 1.0 mmol) and 2,4-dichloro-5-fluoropyri -midine (374 mg, 2.0 mmol) were dissolved in anhydrous alcohol (25 mL) and mixed slowly with DIEA (0.5 mL). The mixture was stirred at 40 ^{o}C for overnight. After the reaction was complete detected by TLC (PE/EA=1:1), the mixture was filtered and the filter cake was washed with 10 mL of EtOH, dried in vacuum to give crude compound **1** (247 mg, 84.7% yield), which was used without purification.

A mixture of compounds **1** (247 mg, 0.85 mmol), (*2R,6S*)-2,6-dimethylpiperazine (484 mg, 4.23 mmol) were dissolved in n-BuOH (10 mL), then DIEA (0.44 mL, 2.55 mmol) was added. The mixture was stirred at 120 ^{o}C for overnight. After the reaction was complete detected by TLC (CH_2Cl_2/MeOH=10:1), the solvent was removed by evaporation. Then the residue was partitioned between CH_2Cl_2 and H_2O. The separated organic layer was washed with water, dried over Na_2SO_4 and evaporated to dryness, and was purified by column chromatography (eluting with PE/EA by 2:1 followed by 100:1−40:1 CH_2Cl_2/MeOH) to give compound WK369 (206 mg, 65.6% yield). ^{1}H NMR (600 MHz, DMSO-d_6) δ 9.65 (s, 1H), 8.10 (dd, J = 5.0, 3.1 Hz, 2H), 8.04 (d, J = 9.5 Hz, 1H), 7.82 (dd, J = 9.0, 2.5 Hz, 1H), 7.42 (d, J = 8.9 Hz, 1H), 6.52 (d, J = 9.5 Hz, 1H), 4.53 (d, J = 12.3 Hz, 2H), 3.24 – 3.20 (m, 2H), 2.87 – 2.77 (m, 2H), 1.25 (d, J = 6.0 Hz, 6H). HRMS (ESI): calcd for $C_{19}H_{21}FN_5O_2$ $[M+H]^+$, 370.1674; found, 370.1670. FX1[1], TAK001[2]and BI3802[3] were synthesized according to the described of literature.

Molecular Docking

A molecular docking study was performed by employing the crystal structures of the BCL6-BTB domain co-crystallized with the ligand pyrazolo-pyrimidine macrocyclic (PDB ID code: 5N1Z) by searching the Protein Data Bank[1] Docking assays were performed by using AutoDock4.2.6[4], and the parameter files and the binding poses were obtained by using AutoDockTool 1.5.6 and the Lamarckian genetic algorithm, respectively. The co-crystallized ligand was used to define the active sites for docking, and the size of the box was 60*60*60. The number of conformational docking simulations was 200, and the lowest binding energy was selected for analysis.

Homogenous Time Resolved Fluorescence (HTRF) Assay

A total volume of 8 μL of BCL6-GST protein and SMRT-6His peptide were added to each well in 384-well plates (Greiner Bio-one, 784045), and then 2 μL of diluent buffer with the compounds to be tested was added. After 1 h, a total volume of 10 μL of anti-6His-XL665 (Cisbio) and anti-GST-Tb (Cisbio) was added to reach a 20 μL final volume. After overnight incubation, the plate was read on a microplate reader (BioTek Cytation5) at 665 nm and 620 nm.

Luciferase reporter assay

The reporter construct (GAL4)5-TK-LUC and the GAL4-DBD-BCL6^{BTB}, GAL4-DBD-Kaiso^{BTB} and GAL4-DBD-PLZF^{BTB} expression plasmid were kindly provided by Dr. Ari Melnick (Weill Cornell Medical College, Department of Haematology/Oncology, New York, NY, USA). A TK-Renilla luciferase plasmid was

included as an internal control (Promega, WA, USA). 293T cells were co-transfected with $(GAL4)_5$-TK-LUC and GAL4-DBD-BCL6^{BTB} or GAL4-DBD-Kaiso^{BTB}, GAL4-DBD-PLZF^{BTB} and GAL4-DBD plus a TK-Renilla reporter construct using Lipofectamine 2000 (Thermo Fisher Scientific). After transfection for 6 h, the cells were treated with the compounds for 24 h. A dual luciferase assay was performed according to the manufacturer's guidelines (Promega, WA, USA).

Surface plasmon resonance assay

Surface plasmon resonance (SPR) analysis was conducted with a Biacore T200 instrument (GE Healthcare) with CM5 sensor chip (GE Healthcare). To test WK369 binding of BCL6-BTB protein, serially diluted concentrations of WK369 were injected into the flow system. Experiments were conducted using phosphate buffered saline (PBS) and the analyte was injected at the flow rate of 30 μL/min. The association time was 60 s and the dissociation time was 30 s. Since WK369 was dissolved in PBS containing 5% dimethyl sulfoxide and a solvent correction assay was performed to adjust the results. BCL6-BTB protein was immobilized on the sensor chip (CM5) using the amine-coupling method according to standard protocols. WK369 at various concentrations were injected into the flow system. The kinetics and affinity assay were examined at 25 °C at a flow rate of 30 μL/min. The K_D values were calculated with the kinetics and affinity analysis option of Biacore 8K evaluation software (version 3.0).

Quantitative Real-Time PCR

PCR primers are listed as follows:

p53: CCCTTCCCAGAAAACCTACC and AATCAACCCACAGCTGCAC,

CDKN1A: CTGAAGGGTCCCCAGGTG and TAGGGCTTCCTCTTGGAGAA,

ATR: GGAGATTTCCTGAGCATGTTCGG and GGCTTCTTTACTCCAGACCA,

CD69: CTGGTCACCCATGGAAGTG and CATGCTGCTGACCTCTGTGT,
GAPDH: GAAGGTGAAGGTCGGAGTC and GAAGATGGTGATGGGATTTC

Animal immunity

The eight-week-old male C57BL/6 mice were immunized with antigen NP_{18}-CGG and each mouse was injected intraperitoneally with 100 μg. Two days later, the mice were treated with 50 mg/kg/d of FX1 or WK369 by ip injection. The mice were euthanized after 12 days of continuous administration, spleen and serum were collected. Flow detection of the proportion of GC-B cells in mice, serum for enzyme linked immunosorbent assay.

Flow Cytometric Analysis

Single-cell suspensions were prepared and red blood cells were lysed by ACK. Antibodies used for staining were PerCP/cy5.5-anti-B220 (Biolegend, 103236), FITC-anti-FAS (BD Pharmingen, 554257), eFluor660-anti-GL7 (Invitrogen, 50-5902-82), PerCP/cy5.5-anti-CD4 (BioLegend, 100434), PE-antiCXCR5 (BioLegend, 145504), and APC-anti-PD1 (BioLegend, 109112). The stained cells were analyzed using a FACS Calibur (BD).

Hematoxylin and Eosin (H&E) staining

The paraffin-embedded paraffin tissue was cut into 4 μm thin sections, immersed in xylene I solution, xylene II solution, xylene and ethanol 1:1 mixture for 10 min, then

immersed in 100%, 95%, 85%, 75% ethanol solution and pure water for 3 min, immersed in hematoxylin dyeing solution for about 10 min. The color was separated with 1% hydrochloric acid ethanol for 10 sec, and then immersed in 75%, 85% and 95% ethanol for 3 min in turn after 30 min in tap water. The tissue sections were stained with eosin for 5 sec, then immersed in 95% ethanol, 100% ethanol, xylene and ethanol 1:1 mixture, xylene I solution, xylene II solution for 5 min, and finally sealed with neutral resin.

Immunohistochemistry (IHC)

The paraffin-embedded paraffin tissue was cut into 4 μm thin sections. After dewaxing, sections were washed with antigen repair solution for 20 min and PBS for 5 min. Tissue sections were soaked in 3% H2O2-methanol solution for 15 min in the dark to remove catalase, and then washed with PBST for three times. Then rinse with distilled water 3 times, 2 min each time. Anti-ki67 rabbit antibody (1:50) was added and incubated overnight at 4 °C. The sections were rinsed again and incubated with the corresponding secondary antibody for 60 min at room temperature, and 3,3 '-diaminbenzidine (DAB) for 5-10 min. Hematoxylin staining was performed. The sections were then rinsed with ddH2O for 10 min, washed with alcohol (75% to 100%), and cleared in xylene. Finally, the sheet is sealed with resin.

Ovarian cancer metastatic mouse model

8-week-old female BALB/c nude mice were obtained from the Animal Center of East China Normal University. All animal experimental protocols were approved by

the Animal Investigation Committee of the Institute of Biomedical Sciences, East China Normal University. ES-2 cells that stably expressed luciferase (ES-2-luc) were embedded in Matrigel (BD Biosciences) and intrasplenically injected into mice. Tumors were allowed to grow for 1 week and treatment of the mice with either compound or control was started after grouping of the mice according to average bioluminescence. The IVIS Imaging System (Xenogen Corporation) was used to monitor pancreatic tumor growth and metastasis. Images and measurements of bioluminescent signals were acquired and analyzed using Living Image and Xenogen software.

Statistical Analyses

Statistical analysis was carried out using Prism 8 (GraphPad Software). Data are represented as the mean ± sd. Statistical comparisons were calculated by One-way ANOVA followed by Tukey's test. $P < 0.05$ was considered statistical significance.

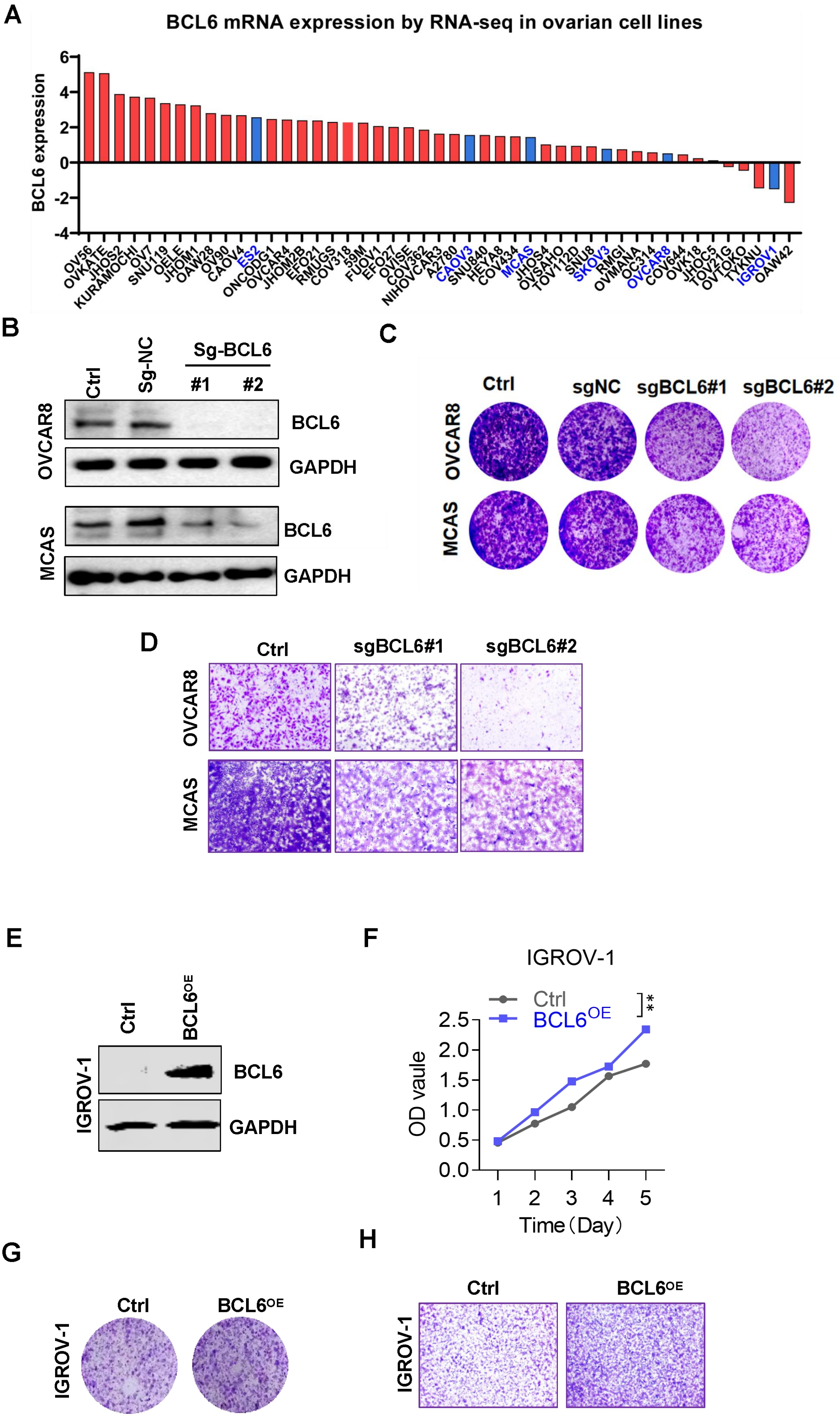

A
BCL6 mRNA expression by RNA-seq in ovarian cell lines
BCL6 expression
6
4
2
0
-2
-4
OV56
OVKATE
JHOS2
KURAMOCHI
OV7
SNU119
OELE
JHOM1
OAW28
OV90
CAOV4
ES2
ONCODG1
OVCAR4
JHOM2B
EFO21
RMUGS
COV318
59M
FUOV1
EFO27
OVISE
COV362
NIHOVCAR3
A2780
CAOV3
SNU840
HEYA8
COV434
MCAS
JHOS4
OVSAHO
TOV112D
SNU8
SKOV3
RMGI
OVMANA
OC314
OVCAR8
COV644
OVK18
JHOC5
TOV21G
OVTOKO
TYKNU
IGROV1
OAW42
B
Ctrl
Sg-NC
Sg-BCL6
#1
#2
OVCAR8
BCL6
GAPDH
MCAS
BCL6
GAPDH
C
Ctrl
sgNC
sgBCL6#1
sgBCL6#2
OVCAR8
MCAS
D
Ctrl
sgBCL6#1
sgBCL6#2
OVCAR8
MCAS
E
Ctrl
BCL6OE
IGROV-1
BCL6
GAPDH
F
IGROV-1
Ctrl
BCL6OE
**
OD vaule
2.5
2.0
1.5
1.0
0.5
0.0
1
2
3
4
5
Time（Day）
G
Ctrl
BCL6OE
IGROV-1
H
Ctrl
BCL6OE
IGROV-1

Figure S1. **BCL6 is overexpressed in Ovarian Cancer and Promotes Ovarian Cancer Proliferation and Metastasis. (A)** Analyze the expression level of BCL6 in ovarian cancer cell lines based on database data. **(B)** The scrambled sgRNA or BCL6 sgRNA1# and 2# was transfected in OVCAR8 and MCAS cells. the knockout efficiency was examined by western blot assays. **(C)** The same series of Ovarian cancer cells were subjected to colony formation assays after 48 h. Colonies were photographed, enumerated, and analyzed after 7 days. **(D)** Transwell assays of sgBCL6 cell lines derived from OVCAR8 and MCAS cells, the number of invasive cells were evaluated after 24 h. **(E)** Efficacy of BCL6 overexpression in IGROV-1 cells by immunoblot analysis. **(F-H)** BCL6 overexpression ($BCL6^{OE}$) enhance IGROV-1 proliferation and migration capabilities indicated by (F) growth curves , (G) relative cell viability of cultured colonies(G) and Transwell assays (H). **P < 0.01, n.s. not significant by log-rank (Mantel–Cox) test.

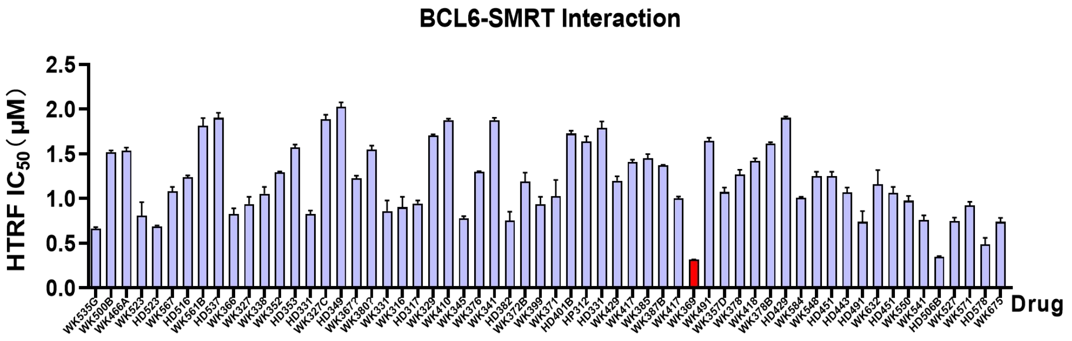

Figure S2. **WK369 is a Novel and Potent of BCL6 Small Molecular Inhibitor.** Candidate compounds obtained through the preliminary screening of the HTRF drug screening system, the red one is WK369.

A

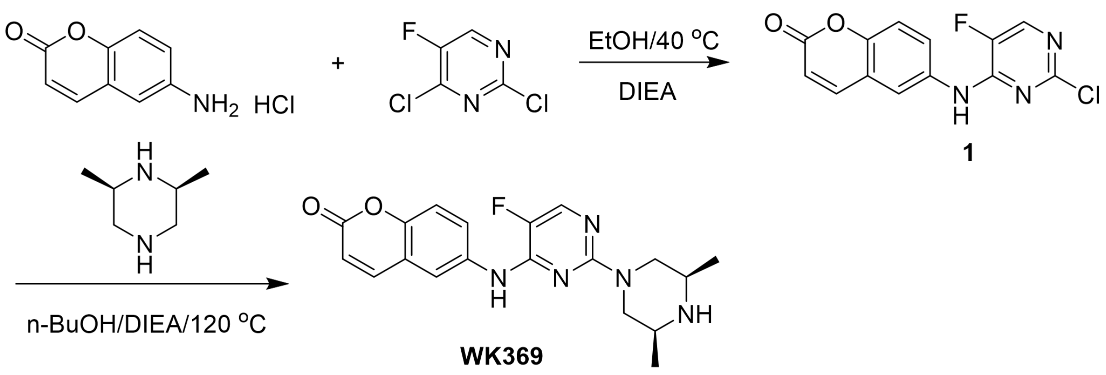

The synthesis scheme of WK369

B

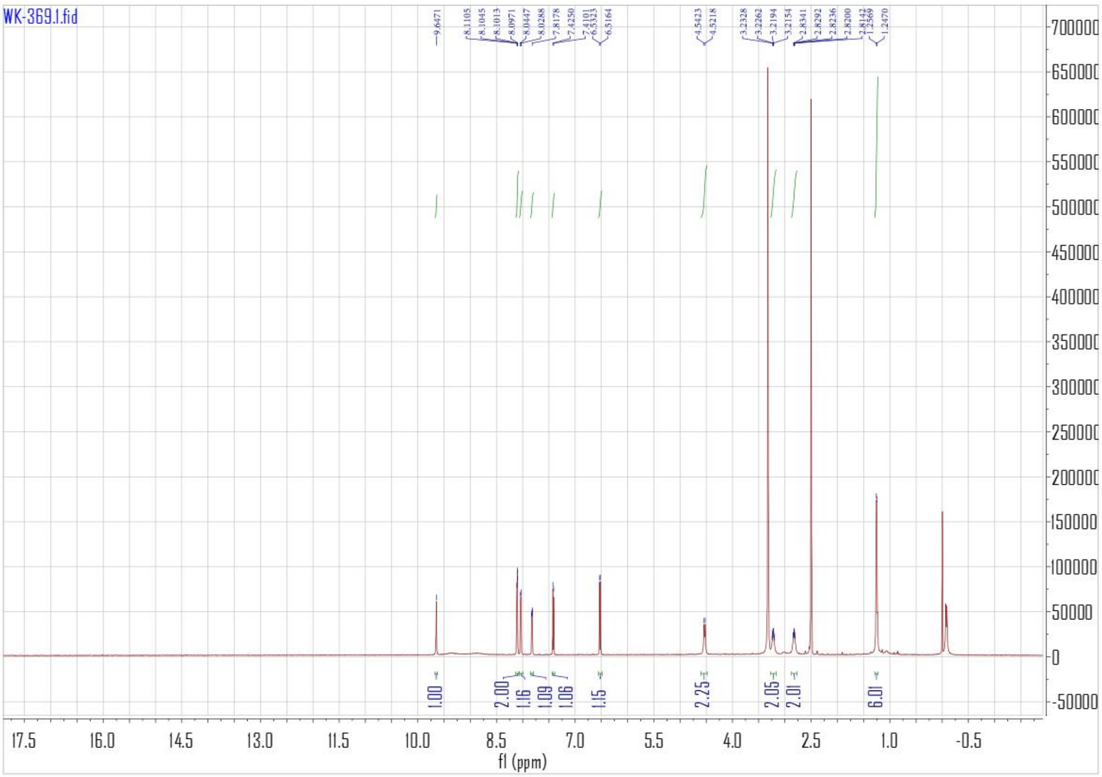

Figure S3. **(A)** The synthesis scheme of WK369. **(B)** ^{1}H NMR spectrum of WK369. NMR spectra were recorded on a Bruker 600 MHz instrument and obtained as DMSO-d_6 solutions (reported in ppm).

Mass Spectrum List Report

Analysis Info

Analysis Name	D:\Data\waixi\chenyihua\WK-369_P1-C-4_01_29544.d	Acquisition Date	11/19/2021 2:58:06 PM
Method	Tune_pos_low_LC with calibration_2min_20210510.m	Operator	ECNU-Chem
Sample Name	WK-369	Instrument	maXis impact 282001.00122
Comment			

Acquisition Parameter

Source Type	ESI	Ion Polarity	Positive	Set Nebulizer	1.5 Bar
Focus	Active	Set Capillary	4500 V	Set Dry Heater	180 °C
Scan Begin	50 m/z	Set End Plate Offset	-500 V	Set Dry Gas	6.0 l/min
Scan End	1350 m/z	Set Collision Cell RF	700.0 Vpp	Set Divert Valve	Waste

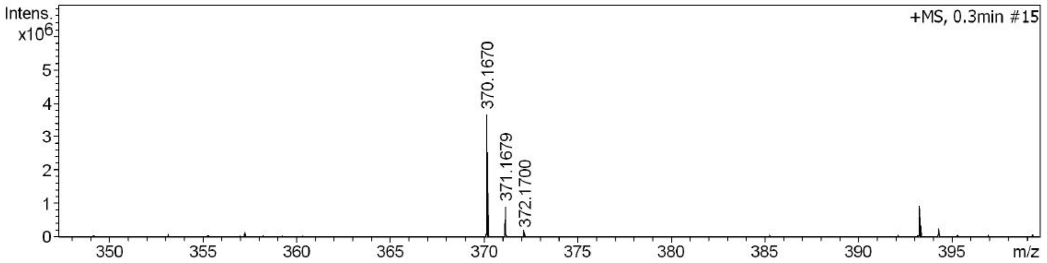

#	m/z	Res.	S/N	I	I %	FWHM
1	370.1670	11645	2492.1	3661776	100.0	0.0318
2	371.1679	21879	627.3	922416	25.2	0.0170
3	372.1700	14882	80.1	117920	3.2	0.0250

Meas. m/z	#	Ion Formula	m/z	err [ppm]	mSigma		Score	rdb	e⁻ Conf	N-Rule
370.1670	1	C19H21FN5O2	370.1674	1.2	14.6	1	100.00	11.5	even	ok

Figure S4. The high-resolution mass spectrum (HR MS) of WK369. High-resolution mass spectrum (HRMS) was gathered on a Bruker MicroTOF-Q II LCMS instrument operating in electrospray ionization (ESI).

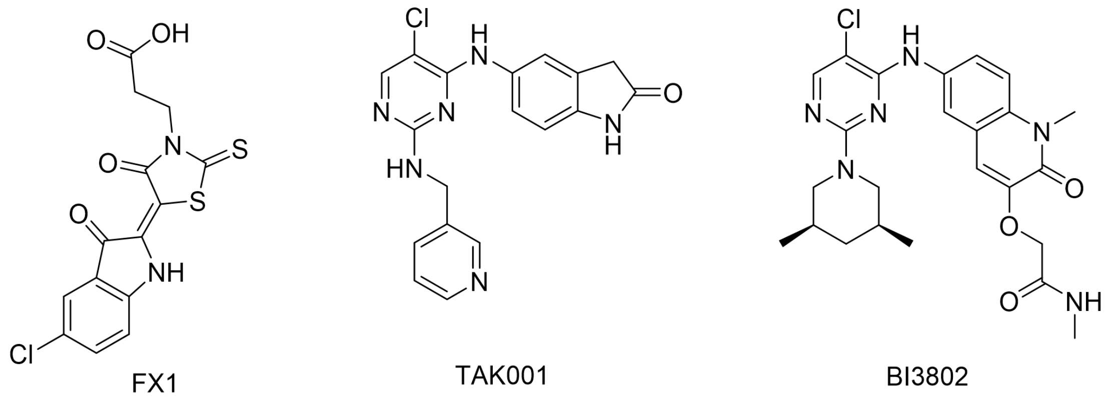

FX1 TAK001 BI3802

Figure S5.**The chemical structure of FX1, TAK001 and BI3802.**

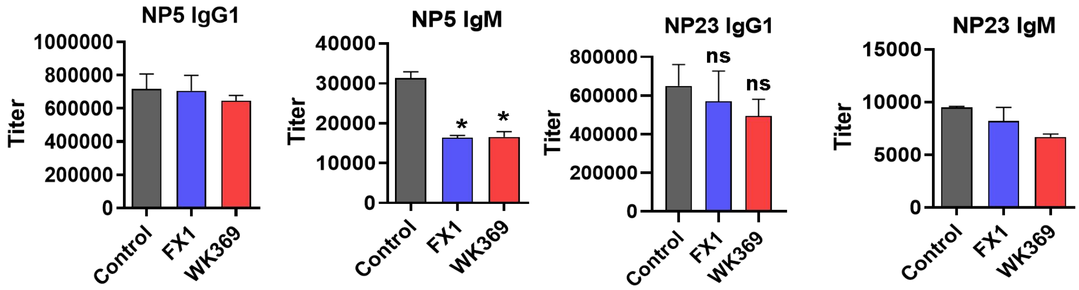

Figure S6. **WK369 suppresses transcriptional inhibitory activity of BCL6 *in vitro* and *in vivo.*** Titers of the NP-specific immunoglobulin G1 antibody and immunoglobulin M antibody measured by ELISA with NP5-BSA and NP23-BSA in the serum from mice immunized with NP-CGG and presented in relative units as serial dilution of the serum relative to antibody end-point titers. Data are expressed as mean ± SD, *P < 0.05, n.s. not significant by oneway ANOVA followed by multiple comparisons.

A

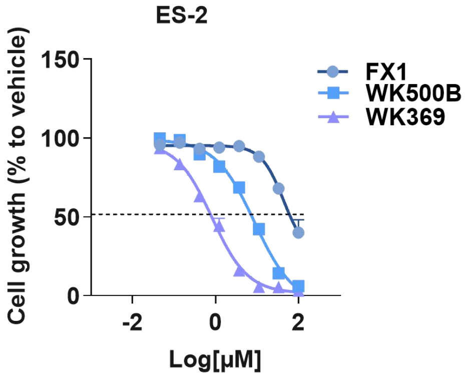

B

Compounds	IC$_{50}$ (μM)
FX1	37.83±0.76
WK500B	8.78±0.32
WK369	1.19±0.26

C

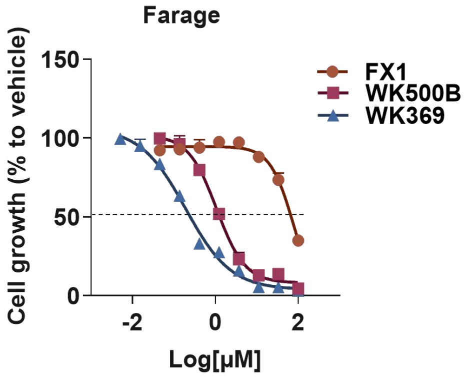

D

Compounds	IC$_{50}$ (μM)
FX1	67.33±1.16
WK500B	1.07±0.02
WK369	0.19±0.01

Figure S7. **WK369 inhibits ovarian cancer cell and DLBCL cancer cell proliferation better than FX1 and WK500B.** The antiproliferation activities of WK369, WK500B and FX1 were determined by an MTS assay in ES-2 and Farage cells.

A

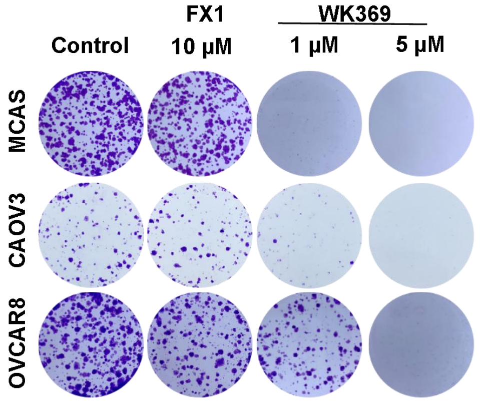

Control
FX1
10 μM
WK369
1 μM
5 μM
MCAS
CAOV3
OVCAR8

B

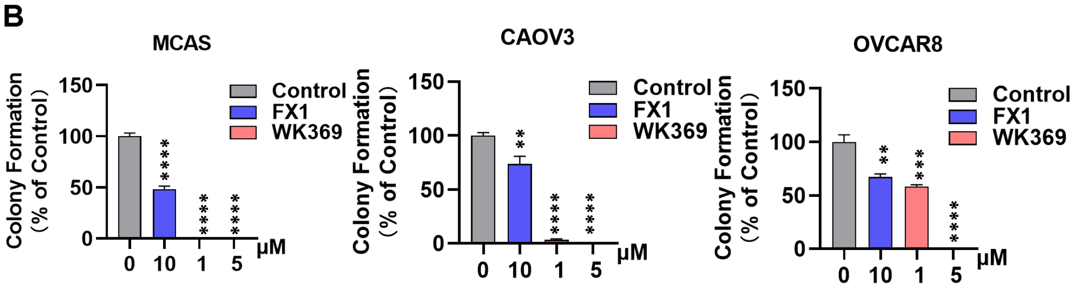

MCAS
CAOV3
OVCAR8
Colony Formation
(% of Control)
150
100
50
0
Control
FX1
WK369
0
10
1
5
μM

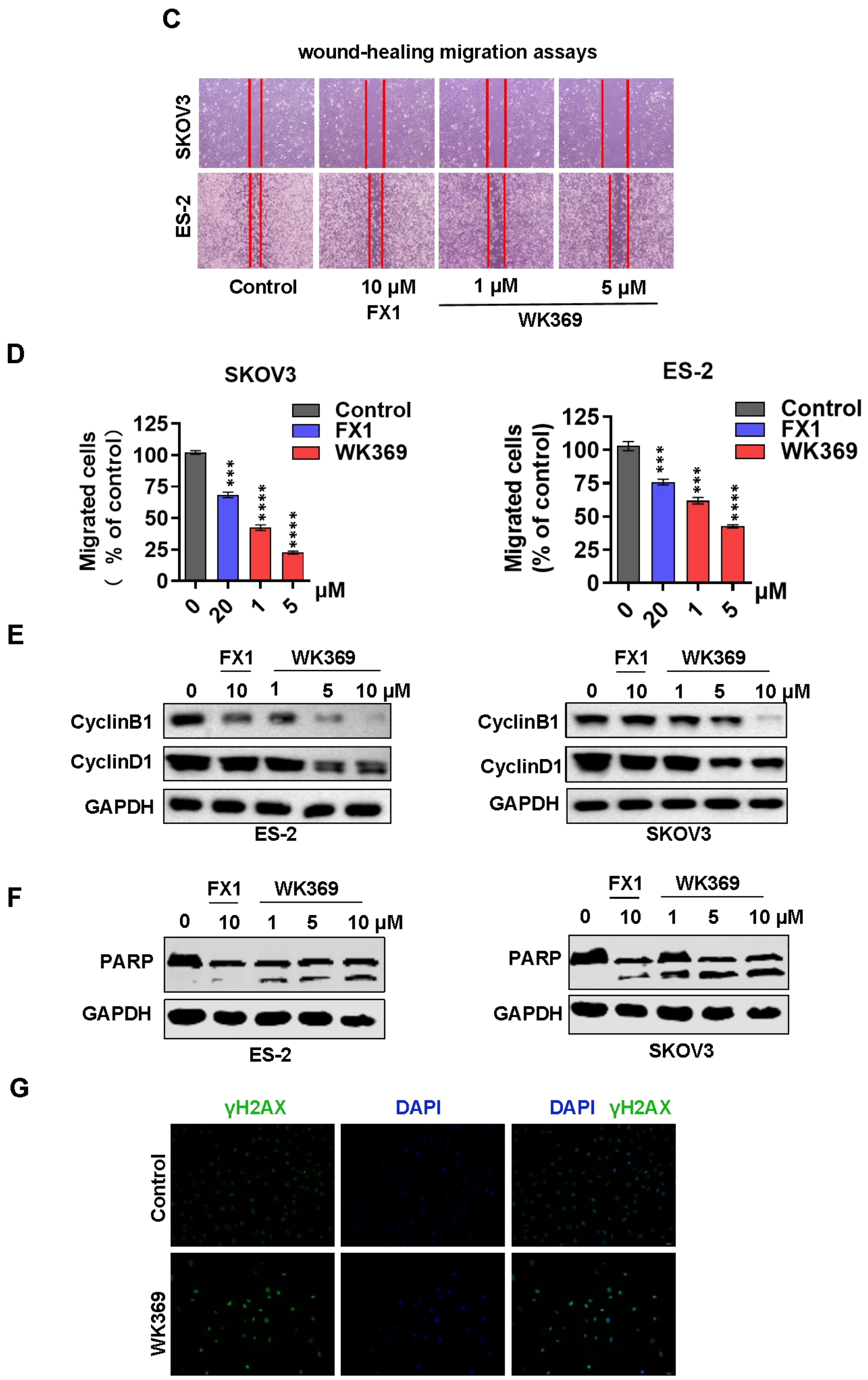

Figure S8. WK369 Suppresses Ovarian Cancer Cells Growth, Migration and Induces Apoptosis. (A-B) WK369 and FX1 inhibited the ovarian cancer cell line (MCAS 、 CAOV3 and OVCAR8) clone formation statistical chart, where the concentration of FX1 was 10 μM and WK369 was 1μM and 5μM. Colonies were

photographed, enumerated, and analyzed after 7 days. **(C-D)** WK369 inhibited ES-2 and SKOV3 migration. ES-2 and SKOV3 cells treated with different concentrations of WK369. After 12 h, the cells that migrated were photographed and quantified. Each bar in the histogram represents means ± SD of three independent experiments. **(E)** Detect the effect of regulating the expression level of G2/M phase related cyclin by western blot assays. **(F)** Detect the effect of apoptosis-related protein expression level by western blot assays. **(G)** Results of immunofluorescence florescent staining for γ H2AX,the cells were treated with WK369. **P < 0.01, ***P < 0.001, ****P < 0.0001, n.s. not significant by log-rank (Mantel–Cox) test.

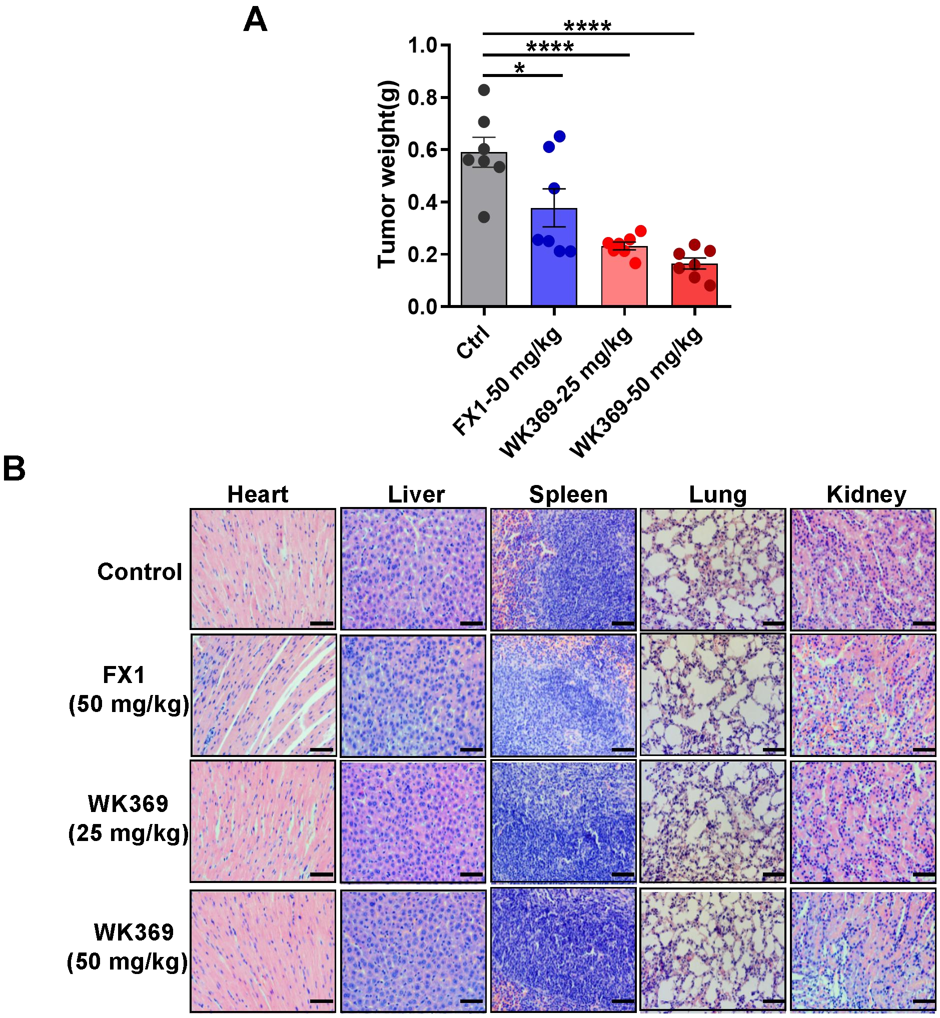
A
****
****
*
1.0
0.8
0.6
0.4
0.2
0.0
Tumor weight(g)
Ctrl
FX1-50 mg/kg
WK369-25 mg/kg
WK369-50 mg/kg
B
Heart
Liver
Spleen
Lung
Kidney
Control
FX1
(50 mg/kg)
WK369
(25 mg/kg)
WK369
(50 mg/kg)

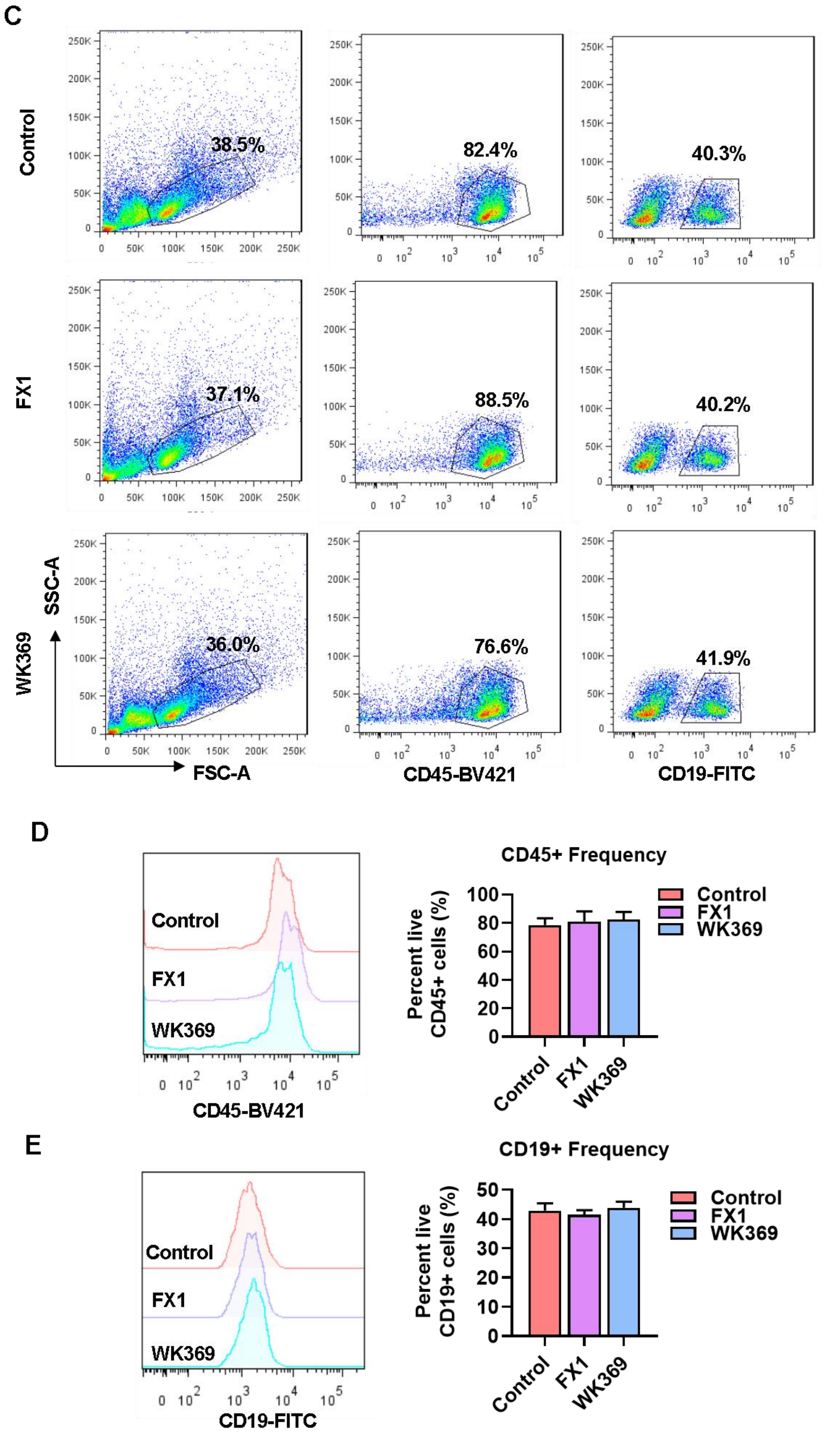
C
Control
FX1
WK369
38.5%
82.4%
40.3%
37.1%
88.5%
40.2%
36.0%
76.6%
41.9%
SSC-A
FSC-A
CD45-BV421
CD19-FITC
D
Control
FX1
WK369
CD45-BV421
CD45+ Frequency
Percent live CD45+ cells (%)
Control
FX1
WK369
E
Control
FX1
WK369
CD19-FITC
CD19+ Frequency
Percent live CD19+ cells (%)
Control
FX1
WK369

Figure S9. **WK369 prevents ovarian cancer growth *in vivo* and no cases of toxic effects were noted in WK369 treated groups.** In a subcutaneous Ovarian Cancer xenograft animal model, after the mice were sacrificed, the tumors were weighed **(A)** and the hearts, livers, spleens, lungs and kidneys from mice were harvested for H&E staining and imaged **(B)**. **(C)** Representative FACS panels for analysis of composition of CD45 and CD19. Representative overlayed histograms **(D)** and bar graphs **(E)** showing the percentages of immune cells and B cell subsets.Scale bars, 50 μm.*P < 0.05,****P < 0.0001, n.s. not significant by log-rank (Mantel–Cox) test.

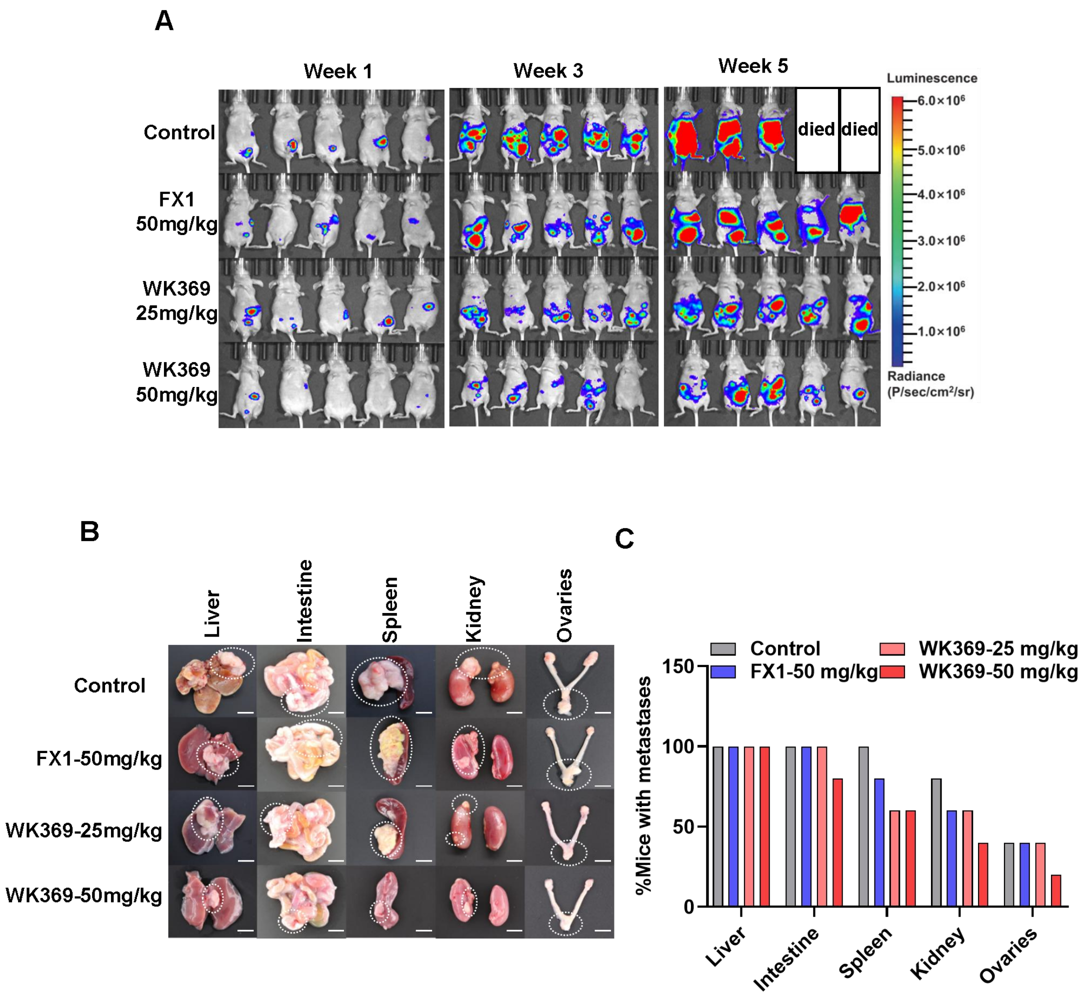

Figure S10. **WK369 Suppress Ovarian Cancer Tumors Intra-abdominal Metastasis *in vivo.* (A-C)** SKOV3 cells were injected subcutaneously into female BALB/c-nude mice. The representative images of tumor metastasis signals by the IVIS Imaging System **(A)**. After 5 weeks, mice were sacrificed and the liver, intestine, spleens, kidney and ovarian from mice were harvested. The tumor of liver, intestine, spleens, kidney and ovarian were photographed and analyzed**(B-C)**. scale bar, 0.5 cm.

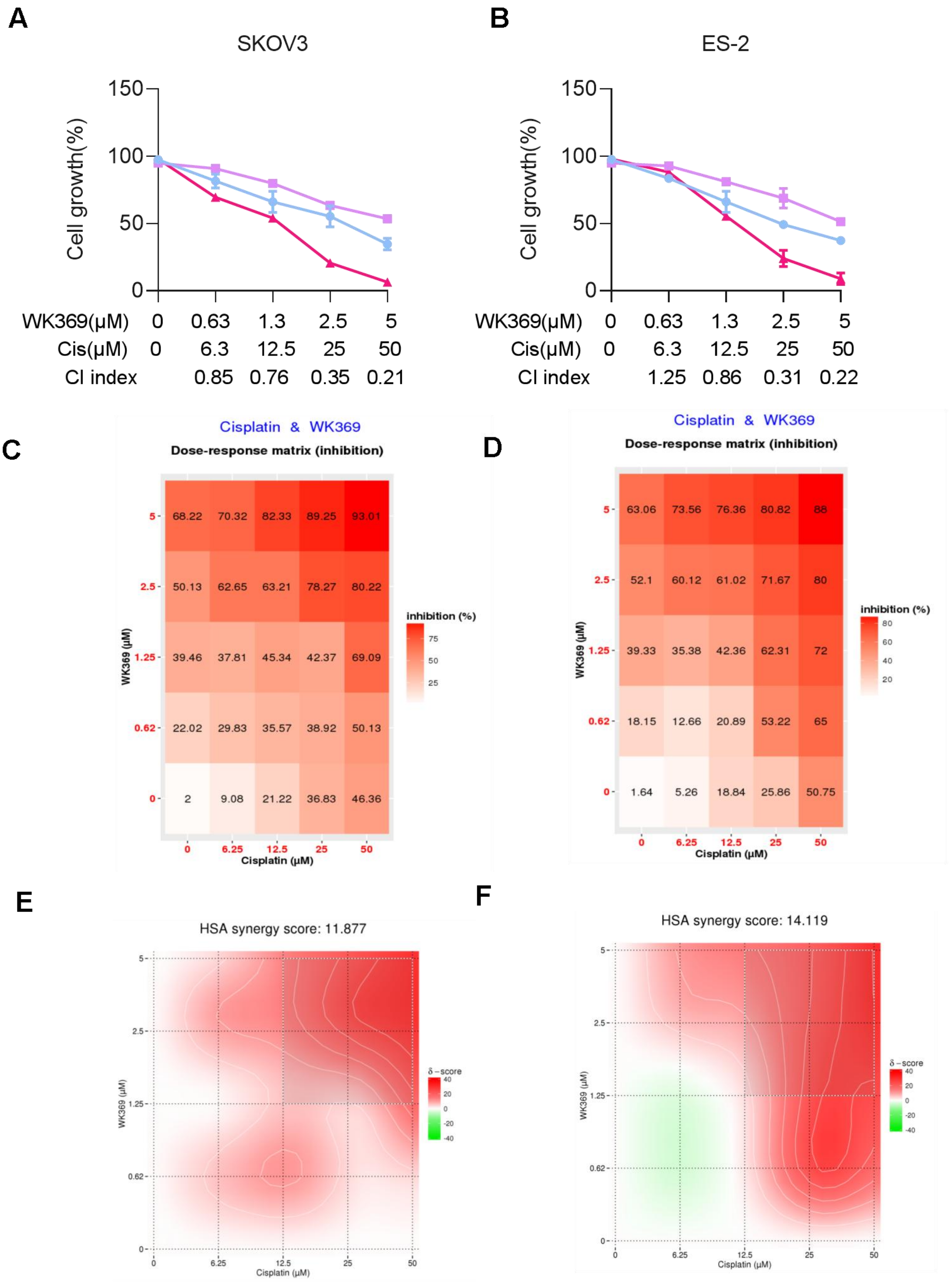

Figure S11. **Effect of WK369 in combination with Cisplatin on ovarian cancer cell growth. (A)** the plots of proliferation after treatment with WK369 and Cisplatin in SKOV3 and ES-2 cell lines. CI ＜1 indicate Synergism.The Inhibition rate after treatment with WK369 and Cisplatin in the SKOV3 and ES-2 cell lines**(C-D),**

Combinational matrix shows proliferation after treatment with WK369 and Cisplatin in the SKOV3 and ES-2 cell lines **(E-F)**. Synergy scores were calculated using Synergyfinder software. HSA (highest single agent) algorithm was used to calculate synergy scores, and color keys indicate the scores in heatmaps.Synergy scores under −10, from −10 to 10 and above 10 indicated antagonism, additivity and synergism.

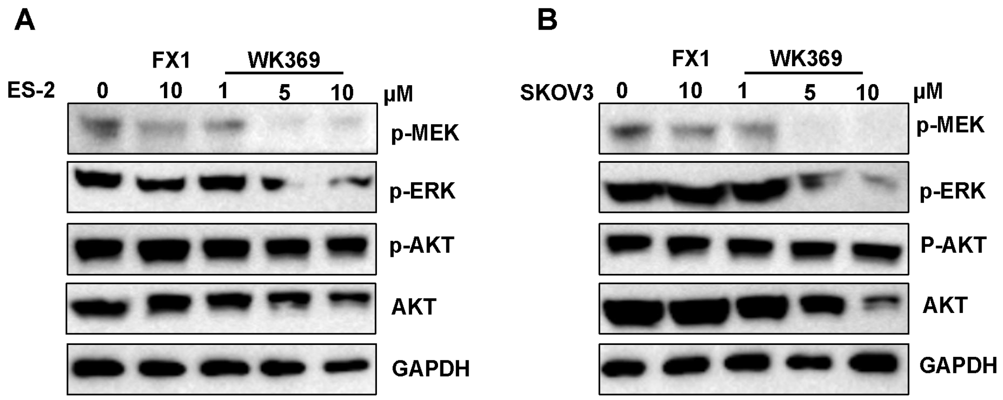

Figure S12. **WK369 Inhibits AKT and MEK/ERK Signaling Pathway by Targeting BCL6. (A-B)** Expression levels of AKT and MER/ERK were detected by western blot following treatment of ES-2 and SKOV3 cells with WK369.

A

PK Parameters of WK369 in Plasma Following IV and PO Administration

PK parameters	Intravenous（IV）	oral gavage（PO）
dose (mg/kg)	1	10
T_{max} (h)	0.08	0.7
C_{max}(ng/mL)	99.2	197
AUC0-$t_{0\text{-}24}$(h*ng/mL)	61.7	464
AUC0-∞ (h*ng/mL)	64.7	468
V_Z(mL/kg)	18131	27424
CL (mL/h/kg)	15727	21390
$MRT_{0\text{-}24}$	0.6	1.6
$t_{1/2}$ (h)	0.8	0.9
F(%)		72.36

Figure S13. Main pharmacokinetic parameters of WK369 after oral administration or intravenous injection in rats(mean ± SD).

Supplemental references

1. Cardenas MG, Yu W, Beguelin W, Teater MR, Geng H, Goldstein RL, et al. Rationally designed BCL6 inhibitors target activated B cell diffuse large B cell lymphoma. *The Journal of clinical investigation* 2016;**126**:3351-62.
2. Kamada Y, Sakai N, Sogabe S, Ida K, Oki H, Sakamoto K, et al. Discovery of a B-Cell Lymphoma 6 Protein-Protein Interaction Inhibitor by a Biophysics-Driven Fragment-Based Approach. *Journal of medicinal chemistry* 2017;**60**:4358-68.
3. Kerres N, Steurer S, Schlager S, Bader G, Berger H, Caligiuri M, et al. Chemically Induced Degradation of the Oncogenic Transcription Factor BCL6. *Cell reports* 2017;**20**:2860-75.
4. Morris GM, Huey R, Lindstrom W, Sanner MF, Belew RK, Goodsell DS, et al. AutoDock4 and AutoDockTools4: Automated docking with selective receptor flexibility. *Journal of computational chemistry* 2009;**30**:2785-91.
